# VARE: Geometry-Anchored Bearing and Range Stabilization for USV Recovery

**DOI:** 10.3390/s26175347

**Published:** 2026-08-24

**Authors:** Chen Chen, Ze Sun, Jiale Zhang, Peng Zhang, Junwei Dong, Run Qian, Dan Wang

**Affiliations:** 1 Taihu Laboratory of Deepsea Technological Science, Wuxi 214082, China; zesun@hnu.edu.cn (Z.S.); zhangjiale@cssrc.com.cn (J.Z.); zhangpeng@cssrc.com.cn (P.Z.); dongjunwei@cssrc.com.cn (J.D.); chan13348106069@stu.xjtu.edu.cn (R.Q.); 2 China Ship Scientific Research Center, Wuxi 214082, China; 3 State Key Laboratory of Integrated Services Networks, Xidian University, Xi’an 710071, China; danwang@xidian.edu.cn

**Keywords:** unmanned surface vehicle (USV), fiducial marker, bearing estimation, range stabilization, RTK-referenced validation, monocular depth, adaptive filtering

## Abstract

Reliable unmanned surface vehicle (USV) recovery requires near-field maritime remote sensing outputs that remain stable during the final approach. Planar fiducial geometry provides metric pose estimates, but its depth channel is sensitive to corner-localization noise, apparent marker shrinkage, glare, reflection, and vessel vibration. We present VARE (Visual-Adaptive Ranging and Estimation), a geometry-anchored perception pipeline that combines ArUco-based Perspective-*n*-Point pose recovery, dual-path bearing fusion, MiDaS-assisted range stabilization, depth-consistency confidence weighting, and innovation-adaptive temporal filtering. VARE is a system-level integration rather than a new neural architecture, PnP solver, or end-to-end docking controller. The pipeline explicitly separates image-centroid bearing, translation-vector bearing, marker-normal heading, camera-frame horizontal approach range, and lateral offset. Independent RTK-synchronized external references, with measured lever-arm corrections between the RTK antenna, camera optical center, and marker reference point, are used for pool and near-shore evaluation. In controlled land tests, VARE reduced independent-reference angular RMSE by 34.0–49.3% relative to the pixel-only baseline and by 22.0–36.4% relative to a static-filter PnP variant. Across 15 pool-based approach trials, the full vision-only configuration achieved a horizontal bearing RMSE of 0.46 degrees, a range MAE of 0.82 m, and a range RMSE of 0.90 m. Relative to the matched IPPE-square geometry baseline with One-Euro filtering, the corresponding descriptive reductions were 14.8%, 4.7%, and 5.3%; the modest range differences are not presented as universally significant. Trial-level summaries, confidence intervals, and data-availability provisions are added to support reproducibility. The results support VARE as a candidate perception module for RTK-referenced USV recovery guidance, while full six-degree-of-freedom validation, session-level dropout survival, and closed-loop capture success remain future work.

## 1. Introduction

Unmanned surface vehicles (USVs) increasingly support survey, monitoring, and logistics tasks in environments that are costly or hazardous for crewed vessels [1,2,3]. Their operational autonomy nevertheless depends on reliable launch and recovery. The final approach is particularly demanding because relative motion, wave disturbance, glare, and nearby structures can degrade both perception and control. This terminal phase also exposes a general field-robotics problem: a perception system may continue to detect a target while the control-relevant cues of centering, lateral convergence, heading, and closing distance disagree.

Recovery guidance requires more than a closing-distance estimate. A controller must determine whether the target is centered in the camera view, whether lateral displacement is decreasing, and whether the vessel is approaching with an acceptable orientation. Global navigation satellite systems provide an external position reference, but near-structure multipath and antenna-to-target offsets complicate terminal relative guidance [4,5]. Radar and LiDAR can complement vision, although their payload, power, resolution, and environmental sensitivities may be restrictive on small USVs [6,7].

A monocular camera observing a planar fiducial marker offers a compact alternative. ArUco detection and a Perspective-*n*-Point (PnP) solver recover a metric pose from known marker geometry [8,9,10,11,12]. The resulting channels do not have equal reliability. Image-centroid bearing depends mainly on calibrated pixel geometry, whereas PnP depth becomes increasingly sensitive to corner perturbations as the apparent marker size decreases [13,14]. Short-lived corner errors can therefore produce depth spikes even when the target remains detectable.

Learning-based monocular depth models provide a complementary cue. MiDaS produces dense relative depth with broad cross-domain generalization [15], but its output lacks the metric scale required for recovery guidance. Metric models such as ZoeDepth and Depth Anything narrow this gap [16,17], yet their maritime deployment and computational cost still require careful validation. For the present system, the defensible role of MiDaS is therefore to stabilize a geometrically anchored range channel rather than to replace metric geometry.

**Figure 1:**
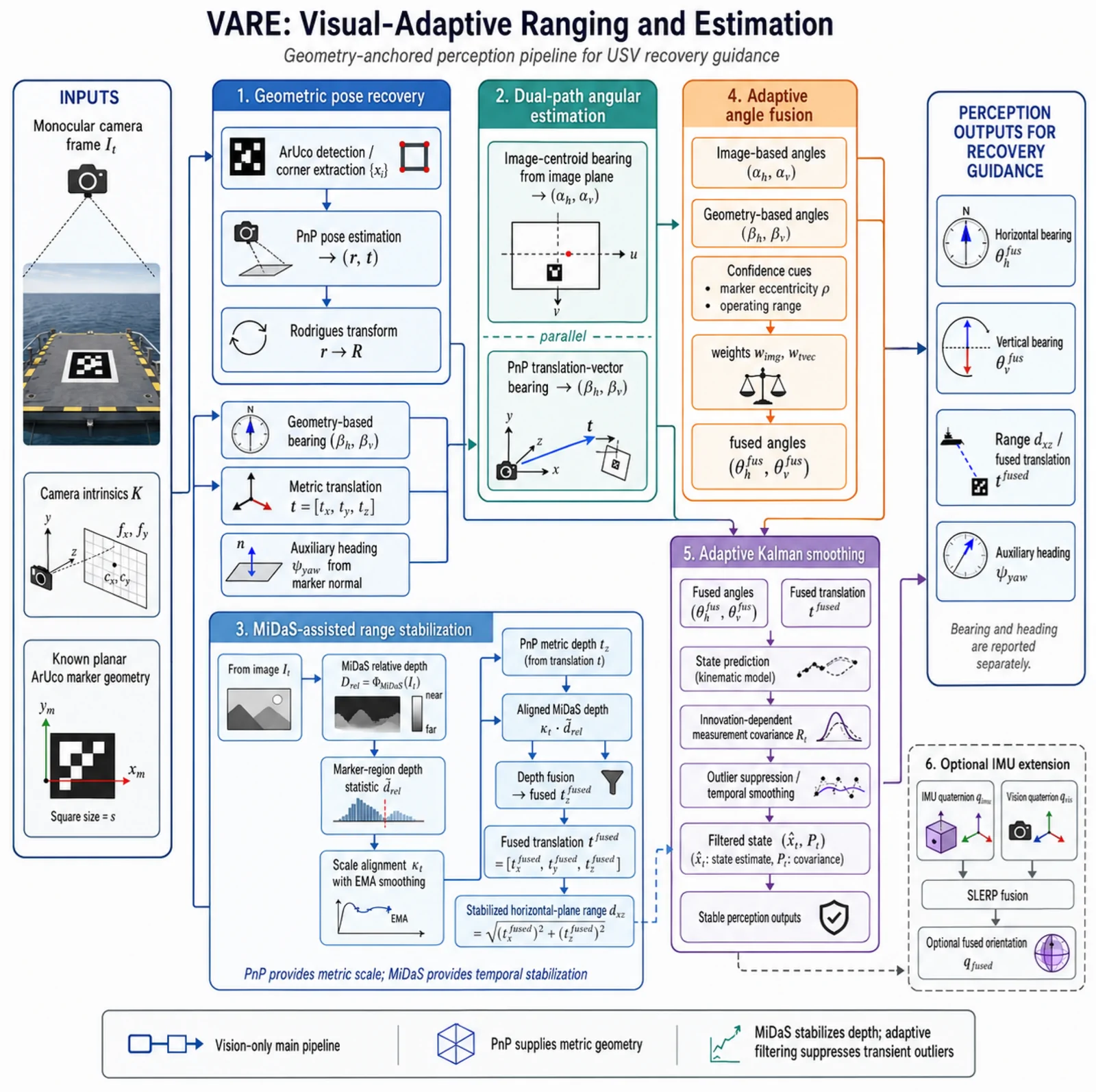
VARE processing pipeline redrawn as a vector schematic. ArUco detections support PnP pose recovery, marker-normal heading extraction, image/PnP dual-path bearing fusion, PnP-anchored MiDaS range stabilization, and innovation-adaptive temporal filtering. The IMU branch is shown only as an optional extension; VARE is a geometry-anchored perception pipeline rather than a depth-network or closed-loop docking autopilot.

We propose VARE (Visual-Adaptive Ranging and Estimation), a vision-only recovery-perception pipeline that separates bearing estimation from range stabilization. VARE combines two bearing paths, aligns MiDaS relative depth to PnP scale, and limits the influence of transient angular residuals through innovation-adaptive covariance. The architecture is shown in Figure 1.

The contributions are system-level and can be tested independently:

1. Dual-path bearing estimation. Image-centroid and PnP-translation bearings are fused with confidence weights based on marker eccentricity and operating range (Section 3.7).
2. Geometry-anchored range stabilization. Relative MiDaS depth is aligned to PnP scale and temporally stabilized, while PnP remains the source of metric scale (Section 3.6.3).
3. Innovation-adaptive temporal filtering. Measurement covariance is increased continuously when the current observation departs strongly from the predicted state, limiting the influence of transient visual outliers without a separate binary acceptance threshold (Section 3.8).
4. Matched evaluation with explicit claim boundaries. Land, pool, river, harbor, and lake tests quantify bearing and externally referenced range error and expose bearing–heading decoupling. A matched IPPE-square baseline and one-at-a-time ablations test whether each integration layer contributes to the recorded output. These comparisons demonstrate measurable, but not uniformly large or super-additive, increments under the recorded conditions; they do not claim full 6-DoF accuracy, state-of-the-art dominance, session-level marker-survival statistics, or closed-loop docking validation.

The scientific hypothesis is therefore architectural and falsifiable. Under identical corner observations and independent references, (i) retaining two differently transformed bearing cues should reduce bearing error relative to disabling their fusion; (ii) PnP-anchored relative-depth stabilization should reduce range error beyond simple smoothing of the same PnP stream; and (iii) innovation-adaptive covariance should provide a smaller additional bearing improvement. The ablations test these questions separately. Their one-at-a-time effects are not interpreted as proof of a factorial interaction or super-additive synergy.

Quaternion-based IMU fusion is retained as an optional extension (Section 3.9) and is excluded from the main vision-only claims.

The remainder of this paper is organized as follows. Section 2 surveys related work in maritime pose estimation, monocular depth inference, and multi-sensor fusion. Section 3 presents the mathematical formulation of each VARE module. Section 4 reports the experimental evaluation across land, pool, and field environments together with ablation studies. Section 6 concludes the paper and outlines future research directions.

## 2. Related Work

### 2.1. Visual Pose Estimation with Fiducial Markers

Planar fiducials replace natural-feature matching with pose estimation from known point correspondences. ArUco [8], AprilTag [18], its flexible-layout AprilTag 3 extension [19], and STag [20] combine marker identification with corner localization. Their pose estimates are commonly recovered with PnP solvers and nonlinear reprojection refinement [9,21,22].

The main limitation for long-range planar-marker guidance is not the absence of a geometric solution, but its sensitivity to image measurements. Corner perturbations can strongly affect translation, particularly the optical-axis component, when the marker occupies few pixels [13,14]. Marker-based USV studies demonstrate the practicality of cooperative visual targets [23], but the available evidence does not establish that one pose channel is uniformly reliable across range and viewing geometry. VARE therefore treats image-domain bearing and PnP translation as separate cues rather than forcing all guidance variables through one pose estimate.

### 2.2. Monocular Depth Estimation

Monocular depth can be inferred from multi-view geometry, known object size, or learned scene priors [24,25,26]. Transformer-based models have improved cross-domain depth prediction [15,27]. MiDaS, however, predicts relative depth and does not independently supply the metric scale needed by a recovery controller. Later models, including ZoeDepth and Depth Anything, extend generalization or metric transfer [16,17], but replacing geometry with a learned metric estimate would require a separate maritime accuracy study. VARE instead uses MiDaS as an auxiliary temporal cue and preserves PnP as the scale anchor.

### 2.3. Sensor Fusion in Marine Robotics

Kalman-family filters provide a common framework for combining uncertain measurements over time [28,29]. Tightly coupled visual–inertial estimators such as VINS-Mono and MSCKF, and visual–inertial SLAM systems such as ORB-SLAM3, estimate ego-motion from feature and inertial constraints [30,31,32]. They address a broader navigation problem than cooperative marker tracking and may impose a larger integration burden on embedded recovery systems.

Fixed-noise filters assume a stationary measurement process. Maritime images violate this assumption when glare, splash, blur, or partial occlusion causes brief error bursts. VARE therefore uses the temporal innovation as a transparent continuous covariance-scaling signal. The optional quaternion interpolation branch is discussed only as a future visual–inertial extension, because the present experiments do not include synchronized inertial logs.

Recent engineering studies also illustrate the value of coordinating heterogeneous information and operational modules. Kweon et al. combined vessel, berth, and weather records to identify demurrage patterns through logical analysis of data [33], while Yoon et al. coordinated truck and autonomous-robot subsystems for heat-wave green-space services [34]. These studies concern decision support and logistics rather than target-relative visual localization, so they are included as system-integration context and not as perception baselines.

### 2.4. Summary of Research Gaps

The literature leaves a practical systems gap. Fiducial geometry supplies metric scale but has range-dependent noise; learned relative depth supplies dense temporal structure but lacks scale; and fixed-noise smoothing does not reflect intermittent maritime outliers. VARE connects these components while keeping their roles explicit. This separation is important because the experiments can test bearing fusion, range stabilization, and continuous innovation adaptation without claiming a new PnP solver or depth network. The present comparison set is designed to isolate these internal roles; it does not replace a future benchmark against stronger docking, AprilTag-board, metric-depth, or visual–inertial pipelines.

## 3. Proposed Method

VARE maps each camera frame to four operational outputs: horizontal and vertical bearing, marker-normal heading, and camera-frame horizontal approach range. The system uses one calibrated monocular camera and a planar ArUco marker with known dimensions. PnP supplies metric geometry, the image plane supplies a distinct but statistically dependent bearing estimate from the same detected corners, and MiDaS supplies a relative depth signal. The experiments evaluate bearing and range error against independent synchronized reference measurements; marker-normal heading is retained as a raw diagnostic variable. They do not validate the complete 6-DoF pose returned by PnP.

### 3.1. Coordinate Frames and Angle Conventions

To avoid ambiguity in the reported orientation quantities, we fix the following conventions.

World frame $F_{W}$. Aligned with the docking infrastructure: $X_{W}$ points to the right of the dock when viewed from the water, $Y_{W}$ points upward (against gravity), $Z_{W}$ points outward from the dock face along the nominal approach direction.

Camera frame $F_{C}$. We adopt the standard OpenCV pinhole convention used by solvePnP: $X_{C}$ points to the right of the image, $Y_{C}$ points downward in the image, and $Z_{C}$ points forward along the optical axis into the scene. Hence, the PnP translation component $t_{z}$ is the optical-axis metric depth, $t_{x}$ is the lateral translation, and $t_{y}$ is the vertical camera-frame translation.

Marker frame $F_{M}$. Attached to the planar ArUco board with its origin at the marker center, $X_{M}$ and $Y_{M}$ spanning the board surface, and $Z_{M}$ normal to the board (outward). This center-origin definition makes the PnP translation vector t directly represent the marker-center position in the camera frame.

Operational orientation convention. The rotation matrix R∈SO(3) returned by solvePnP maps marker-frame points into the camera frame, $X_{C}=RX_{M}+t$. We report one marker-normal heading variable, $\psi_{\mathrm{yaw}}$, from the marker normal in the camera frame. We do not interpret an Euler triplet because no full orientation benchmark is available.

Throughout the paper, bearing ($\alpha_{h}$, $\beta_{h}$, $\theta_{h}^{\mathrm{fus}}$) refers to the line-of-sight angle between the marker center and the camera optical axis in the image plane. It is not identical to the marker-normal heading variable $\psi_{\mathrm{yaw}}$. Bearing is zero when the marker projects onto the image principal point. By contrast, $\psi_{\mathrm{yaw}}$ is zero only when the marker normal is parallel to the camera forward axis. This distinction is central to the docking-alignment analysis in Section 4.4. Table 1 summarizes the main reported variables and their operational roles.

### 3.2. Camera Projection Model

The spatial relationship between a 3D object-frame point and its 2D image projection is modeled using the classical pinhole camera model:

(1) $$\lambda_{i}\left[\begin{array}{c} u\\ v\\ 1 \end{array}\right]=K\left[\begin{array}{cc} R & t \end{array}\right]\left[\begin{array}{c} X\\ Y\\ Z\\ 1 \end{array}\right],$$

where $\lambda_{i}$ is the homogeneous depth scale for corner *i*, (u,v) are its pixel coordinates, and (X,Y,Z) are its known marker-frame coordinates. The intrinsic matrix is

(2) $$K=\left[\begin{array}{ccc} f_{x} & 0 & c_{x}\\ 0 & f_{y} & c_{y}\\ 0 & 0 & 1 \end{array}\right],$$

where $f_{x}$ and $f_{y}$ are focal lengths in pixels, and $\left(c_{x},c_{y}\right)$ is the principal point. The extrinsic parameters satisfy $X_{C}=RX_{M}+t$. For the square marker used here, the 3D object points are defined with the marker center as the origin, namely ${\left[-L/2,-L/2,0\right]}^{⊤}$, ${\left[L/2,-L/2,0\right]}^{⊤}$, ${\left[L/2,L/2,0\right]}^{⊤}$, and ${\left[-L/2,L/2,0\right]}^{⊤}$. Under this convention, t is the marker-center translation rather than a corner translation. Mapping these camera-frame quantities into $F_{W}$ requires the calibrated marker-to-infrastructure transform.

### 3.3. PnP-Based Pose Recovery

Given n≥4 known 3D marker corner points $X_{i}={\left[X_{i},Y_{i},Z_{i}\right]}^{⊤}$ and their corresponding detected 2D image projections $x_{i}={\left[u_{i},v_{i}\right]}^{⊤}$, the PnP problem seeks the rotation R and translation t satisfying

(3) $$x_{i}=\pi \;\left(RX_{i}+t\right),\;i=1,\ldots ,n,$$

where π(·) denotes the perspective projection incorporating K. The accuracy of the recovered pose is assessed via the mean reprojection error:

(4) $$E_{\mathrm{reproj}}=\frac{1}{n}\sum_{i=1}^{n}{∥x_{i}-{\hat{x}}_{i}∥}_{2},$$

where ${\hat{x}}_{i}$ is the reprojected corner. We use OpenCV’s SOLVEPNP_ITERATIVE implementation, which refines the pose by minimizing reprojection error [35]. Reprojection error is defined here as a diagnostic, but it is not included in the present confidence weights.

### 3.4. Rotation Representation and Auxiliary Heading Extraction

The PnP solver returns a Rodrigues vector $r\in R^{3}$. For θ=∥r∥>0, define u=r/θ. The rotation matrix is

(5) $$R=I+\sin\theta \;{\left[u\right]}_{\times }+\left(1-\cos\theta \right)\;{\left[u\right]}_{\times }^{2},$$

where ${\left[u\right]}_{\times }$ is the skew-symmetric matrix of u. When θ is numerically zero, we set R=I. Let $e_{z,M}={\left[0,0,1\right]}^{⊤}$ denote the marker normal. Its camera-frame representation is

(6) $$n_{C}=R\;e_{z,M}={\left[R_{13},\;R_{23},\;R_{33}\right]}^{⊤}.$$

We then define the marker-normal heading variable reported throughout the experiments as the horizontal deviation of this normal in the $X_{C}$–$Z_{C}$ plane:

(7) $$\psi_{\mathrm{yaw}}=atan2\;\left(R_{13},\;R_{33}\right).$$

This definition directly matches the physical quantity needed for alignment: $\psi_{\mathrm{yaw}}=0$ when the marker normal is parallel to the camera optical axis, and $|\psi_{\mathrm{yaw}}|$ grows as the vessel points more obliquely at the target. We intentionally avoid assigning controller-level meaning to a full Euler triplet, because doing so would require an additional axis-order convention that is not experimentally validated in the present manuscript. The reported $\psi_{\mathrm{yaw}}$ is therefore a marker-normal heading variable for alignment, not a claim of full 6-DoF orientation benchmarking.

### 3.5. Dual-Path Angular Estimation

Bearing and range respond differently to marker-localization error. Image-centroid bearing depends directly on calibrated pixel offsets. PnP translation additionally depends on the apparent marker geometry and becomes less stable as the marker footprint shrinks [13,14]. VARE therefore retains two bearing paths rather than deriving every angular output from one translation estimate.

We therefore compute angular offsets with two distinct, complementary estimators derived from the same detected corner set. They are not statistically independent: common corner shifts and calibration error can affect both outputs.

Path 1: image-based angles. The horizontal and vertical offsets are

(8) $$\begin{array}{cc} \alpha_{h} & =\arctan\;\left(\frac{u-c_{x}}{f_{x}}\right),\; \end{array}$$

(9) $$\begin{array}{cc} \alpha_{v} & =\arctan\;\left(\frac{v-c_{y}}{f_{y}}\right). \end{array}$$

This path does not require 3D reconstruction. Under the OpenCV convention, positive $\alpha_{v}$ denotes a marker center below the principal point.

Path 2: translation-vector angles. The center-origin PnP translation gives

(10) $$\begin{array}{cc} \beta_{h} & =atan2(t_{x},\;t_{z}), \end{array}$$

(11) $$\begin{array}{cc} \beta_{v} & =atan2(t_{y},\;t_{z}). \end{array}$$

This path preserves the estimated 3D line of sight to the marker center. Positive $\beta_{v}$ follows the same downward-positive convention as $\alpha_{v}$. The angles $\beta_{h}$ and $\beta_{v}$ are computed from the raw center-origin PnP vector before the range-only MiDaS rescaling in Section 3.6.3; uniform range rescaling therefore does not change the geometric line-of-sight angle itself.

The image-centroid estimator compresses the four corners to their positional centroid, whereas PnP additionally uses the quadrilateral shape, known three-dimensional marker geometry, and calibrated projection model. Consequently, the estimators share common-mode corner error but can respond differently to perspective deformation, nonuniform corner perturbation, and depth instability. A first-order horizontal perturbation analysis makes these different transfer functions explicit:

(12) $$\begin{array}{cc} \delta \alpha_{h} & \approx \frac{\delta u}{f_{x}\;\left[1+{\left(\left(u-c_{x}\right)/f_{x}\right)}^{2}\right]}, \end{array}$$

(13) $$\begin{array}{cc} \delta \beta_{h} & \approx \frac{t_{z}\;\delta t_{x}-t_{x}\;\delta t_{z}}{t_{x}^{2}+t_{z}^{2}}. \end{array}$$

Here, δu is the centroid perturbation induced by the shared corner observations, whereas $\delta t_{x}$ and $\delta t_{z}$ are the corresponding PnP translation perturbations. The first path responds directly to centroid displacement; the second also reflects shape-dependent pose coupling. These equations explain complementarity without assuming independence.

If the signed path errors are $e_{\alpha }$ and $e_{\beta }$ and the image-path share after normalization is λ, the fused-error variance is

(14) $$\mathrm{Var}\left(e_{\mathrm{fus}}\right)=\lambda^{2}\sigma_{\alpha }^{2}+{\left(1-\lambda \right)}^{2}\sigma_{\beta }^{2}+2\lambda \left(1-\lambda \right)\mathrm{Cov}\left(e_{\alpha },e_{\beta }\right).$$

Equation (14) makes the claim boundary explicit: fusion cannot cancel a fully common bias and yields additional information only when the two transformations provide non-redundant error components. The retained archive does not contain time-aligned signed frame-level errors for the two paths, so neither Pearson nor Spearman residual correlation can be reconstructed. We therefore report the analytical dependence structure and the matched ablation result, but do not substitute a correlation of grouped RMSE magnitudes for the requested frame-level error correlation.

### 3.6. Geometry-Anchored Range Stabilization

The range channel combines a metric PnP estimate with an aligned relative-depth cue. Marker-size and angular-size estimates are retained as diagnostic geometric checks when a valid marker footprint is available.

#### 3.6.1. Marker-Size-Based Depth

When the physical marker side length *L* is known and its apparent image size is $s_{\mathrm{px}}$ pixels, depth is estimated as

(15) $$z=\frac{f\cdot L}{s_{\mathrm{px}}}.$$

This estimate is available only when the marker extent can still be measured. It is not a fallback after complete detection loss.

#### 3.6.2. Angular-Size-Based Distance

The distance can alternatively be derived from the marker’s apparent angular size $\theta_{\mathrm{app}}$:

(16) $$d=\frac{L}{2\tan\;\left(\theta_{\mathrm{app}}/2\right)}.$$

#### 3.6.3. MiDaS-Assisted Metric Depth Alignment

PnP depth can fluctuate when the detected corners move under blur, vibration, or small marker footprints. MiDaS is used as an auxiliary temporal cue while PnP retains responsibility for metric scale.

Given an input RGB image $I\in R^{H\times W\times 3}$, MiDaS produces a relative (scale-invariant) depth map:

(17) $$D_{\mathrm{rel}}=\Phi_{\mathrm{MiDaS}}\left(I\right),$$

where $\Phi_{\mathrm{MiDaS}}\left(\cdot \right)$ denotes the pretrained encoder–decoder network. Within the region of interest (ROI) defined by the detected marker corners, the representative relative depth is computed as the median to reject local outliers:

(18) $${\tilde{d}}_{\mathrm{rel}}=\mathrm{median}\;\left\{D_{\mathrm{rel}}\left(x,y\right)|\left(x,y\right)\in \mathrm{ROI}\right\}.$$

Scale recovery with temporal persistence. Let $t_{z}$ denote PnP optical-axis metric depth. The instantaneous alignment factor is

(19) $${\hat{\kappa }}_{t}=\frac{t_{z}}{{\tilde{d}}_{\mathrm{rel}}+\epsilon },$$

where $\epsilon =10^{-6}$. The alignment factor is smoothed as

(20) $$\kappa_{t}=\eta \;{\hat{\kappa }}_{t}+\left(1-\eta \right)\;\kappa_{t-1},$$

where η∈(0,1] and η=0.3 in the reported experiments. The aligned MiDaS depth is

(21) $$d_{\mathrm{midas}}=\kappa_{t}{\tilde{d}}_{\mathrm{rel}}.$$

Depth fusion. The fused metric depth combines both sources via a tunable weight $\alpha_{z}\in \left[0,1\right]$:

(22) $$t_{z}^{\mathrm{fused}}=\alpha_{z}\;t_{z}+\left(1-\alpha_{z}\right)\;d_{\mathrm{midas}}.$$

For range-channel reporting, the lateral components are rescaled proportionally to maintain a coherent stabilized translation vector:

(23) $$t^{\mathrm{fused}}=\frac{t_{z}^{\mathrm{fused}}}{t_{z}+\epsilon }\;t.$$

PnP provides the metric anchor that MiDaS lacks. The MiDaS branch is therefore a geometry-anchored range stabilizer, not an independent metric sensor. Equation (23) is used for the reported range and lateral-offset outputs; the translation-vector bearing in Equations (10) and (11) remains defined by the raw center-origin PnP vector. Consequently, MiDaS can influence the final bearing only indirectly through depth-dependent confidence weighting, not by rotating the PnP line-of-sight vector.

Failure mode. The alignment factor $\kappa_{t}$ depends on PnP depth. It becomes stale during prolonged detection loss, and the aligned MiDaS output then loses metric validity. Marker-size estimates also require a measurable marker footprint. Complete marker loss therefore provides no new visual measurement. Illumination or reflection changes can perturb the MiDaS ROI and reduce $q_{z}$ even when PnP remains valid, thereby suppressing a valid geometry contribution. Conversely, severe common image degradation can affect both marker corners and the relative-depth ROI, so the two branches do not guarantee correction of common-mode error.

#### 3.6.4. 3D Euclidean and Horizontal-Plane Distance

The full slant range and camera-frame horizontal approach distance are computed from the fused translation:

(24) $$\begin{array}{cc} d_{xyz} & =∥t^{\mathrm{fused}}{∥}_{2}, \end{array}$$

(25) $$\begin{array}{cc} d_{xz} & =\sqrt{{t_{x}^{\mathrm{fused}}}^{2}+{t_{z}^{\mathrm{fused}}}^{2}}. \end{array}$$

The reported range is $d_{xz}$ because the present evaluation concerns horizontal approach geometry. This choice excludes vertical displacement rather than filtering it statistically.

#### 3.6.5. Docking Alignment Criterion

The outputs of the distance and angle estimation modules are jointly interpreted to determine the alignment state of the USV relative to the docking target. Specifically, successful alignment for recovery requires three conditions to be satisfied simultaneously:

(26) $$\theta_{h}^{\mathrm{fus}}\to 0,\;\psi_{\mathrm{yaw}}\to 0,\;\mathrm{and}\;t_{x}^{\mathrm{fused}}\to 0,$$

where $\theta_{h}^{\mathrm{fus}}$ is the fused horizontal bearing angle (Section 3.7), $\psi_{\mathrm{yaw}}$ is the marker-normal heading variable (Equation (7)), and $t_{x}^{\mathrm{fused}}$ is the lateral component of the fused translation vector (Equation (23)). The first condition enforces camera-line-of-sight centering with respect to the marker. The second condition enforces orientation alignment, so that the marker normal is parallel to the camera forward axis. The third condition ensures that the USV is centered on the docking ramp rather than approaching from a lateral offset. The remaining quantity $d_{xz}$ (Equation (25)) provides the closing distance to the docking structure.

In practice, a controller requires tolerance bands, $|\theta_{h}^{\mathrm{fus}}|<\theta_{\mathrm{tol}}$, $|\psi_{\mathrm{yaw}}|<\psi_{\mathrm{tol}}$, and $|t_{x}^{\mathrm{fused}}|<\delta_{\mathrm{tol}}$, together with a terminal range condition. The field analysis uses 3° as a retrospective bearing tolerance, but it does not validate closed-loop values for $\psi_{\mathrm{tol}}$, $\delta_{\mathrm{tol}}$, or the terminal range threshold.

### 3.7. Adaptive Angle Fusion

The image-based angles $\left(\alpha_{h},\alpha_{v}\right)$ and geometry-based angles $\left(\beta_{h},\beta_{v}\right)$ are fused via confidence-aware adaptive weighting.

#### 3.7.1. Confidence Metrics

The image-path weight uses marker eccentricity as a heuristic reliability proxy:

(27) $$\rho =\frac{1}{\sqrt{2}}\sqrt{{\left(\frac{u-c_{x}}{W/2}\right)}^{2}+{\left(\frac{v-c_{y}}{H/2}\right)}^{2}},$$

Here, ρ=0 at the image center and ρ≤1 inside the image rectangle. The geometry-path weight combines two confidence cues: (i) the agreement between the PnP metric depth and the MiDaS-aligned depth, and (ii) the proximity of the current stabilized range cue to a nominal operating range $z_{\mathrm{ref}}$:

(28) $$\begin{array}{cc} q_{z} & =\exp\;\left(-\frac{|t_{z}-d_{\mathrm{midas}}|}{\tau_{z}}\right), \end{array}$$

(29) $$\begin{array}{cc} w_{\mathrm{img}} & =\max(0.3,\;1-\rho ), \end{array}$$

(30) $$\begin{array}{cc} w_{\mathrm{tvec}} & =q_{z}\exp\;\left(-\frac{|z_{\mathrm{conf}}-z_{\mathrm{ref}}|}{\tau_{r}}\right), \end{array}$$

where $q_{z}$ is a continuous depth-consistency factor, $z_{\mathrm{ref}}$ is the center of the nominal operating-range prior, and $\tau_{z},\tau_{r}>0$ are 1/e decay scales for depth disagreement and range departure, respectively. The confidence depth is set to $z_{\mathrm{conf}}=t_{z}^{\mathrm{fused}}$ after the first valid MiDaS latch and to $z_{\mathrm{conf}}=t_{z}$ before that latch; $q_{z}$ is set to one before a valid MiDaS-aligned depth is available. The floor in $w_{\mathrm{img}}$ prevents the image path from vanishing. These weights are fixed heuristic reliability scores rather than calibrated probabilities.

The parameters have transparent analytical effects summarized in Table 2. The default $\tau_{z}=\tau_{r}=15$ m gives the stated 1/e decay at a 15 m discrepancy or range departure. The image-weight floor becomes active when ρ≥0.7; because $w_{\mathrm{tvec}}\leq 1$, its default value of 0.3 guarantees a normalized image-path share of at least 0.3/(1+0.3)=23.1% when both paths are available.

Only $\alpha_{z}$ is supported by an empirical performance sweep in the present archive (Section 4.5.2). A factorial trial-level sweep of $z_{\mathrm{ref}}$, $\tau_{z}$, $\tau_{r}$, and the image-weight floor is not available; Table 2 is therefore reported as analytical shaping sensitivity rather than empirical robustness evidence.

#### 3.7.2. Fused Angles

The final bearing estimates are the confidence-weighted averages:

(31) $$\begin{array}{cc} \theta_{h}^{\mathrm{fus}} & =\frac{w_{\mathrm{img}}\;\alpha_{h}+w_{\mathrm{tvec}}\;\beta_{h}}{w_{\mathrm{img}}+w_{\mathrm{tvec}}}, \end{array}$$

(32) $$\begin{array}{cc} \theta_{v}^{\mathrm{fus}} & =\frac{w_{\mathrm{img}}\;\alpha_{v}+w_{\mathrm{tvec}}\;\beta_{v}}{w_{\mathrm{img}}+w_{\mathrm{tvec}}}. \end{array}$$

The geometry path receives its largest weight when the PnP and MiDaS-aligned depth cues agree and the stabilized range remains close to $z_{\mathrm{ref}}$. Both depth disagreement and large departure from the nominal operating range shift relative weight toward the image path. Because $z_{\mathrm{conf}}$ and $q_{z}$ come from the range-stabilization branch, parameters such as $\alpha_{z}$ can change the final fused bearing through the weighting term even though they do not alter the raw PnP bearing $\beta_{h}$ or $\beta_{v}$. Reprojection error, marker area, and blur are not used in the current rule.

### 3.8. Adaptive Kalman Filter for Temporal Smoothing

Each fused bearing channel is smoothed by an independent scalar Kalman filter. The measurement noise increases with squared innovation:

Prediction:

(33) $$\begin{array}{cc} {\hat{x}}_{t|t-1} & ={\hat{x}}_{t-1}, \end{array}$$

(34) $$\begin{array}{cc} P_{t|t-1} & =P_{t-1}+Q, \end{array}$$

where *Q* models inter-frame angular variation. Filtering is performed in degrees, and the parameters in Table 5 are therefore in deg^2^. The filter is initialized with ${\hat{x}}_{0}=y_{0}$ and $P_{0}=1.0$ deg^2^ at the first valid detection.

Innovation-adaptive update:

(35) $$\begin{array}{cc} R_{t} & =\max\;\left(R_{\min},\;\gamma \;{\left(y_{t}-{\hat{x}}_{t|t-1}\right)}^{2}\right), \end{array}$$

(36) $$\begin{array}{cc} K_{t} & =\frac{P_{t|t-1}}{P_{t|t-1}+R_{t}}, \end{array}$$

(37) $$\begin{array}{cc} {\hat{x}}_{t} & ={\hat{x}}_{t|t-1}+K_{t}\;\left(y_{t}-{\hat{x}}_{t|t-1}\right), \end{array}$$

(38) $$\begin{array}{cc} P_{t} & =\left(1-K_{t}\right)\;P_{t|t-1}. \end{array}$$

Here, $y_{t}$ is the fused bearing measurement, $\nu_{t}=y_{t}-{\hat{x}}_{t|t-1}$ is the innovation, γ>0 is dimensionless, and $R_{\min}$ is the noise floor. A large innovation increases $R_{t}$ continuously and reduces the Kalman gain. There is no separate binary innovation threshold and no range-hold decision in the evaluated formulation; the earlier wording in Algorithm 1 incorrectly implied one and has been removed. With the reported $R_{\min}=0.1$ deg^2^ and γ=2, the covariance rule changes branch at $|\nu_{t}|=\sqrt{R_{\min}/\gamma }=0.224{}^{\circ}$. This is an analytical crossover of Equation (35), not an accept/reject threshold.
**Algorithm 1** VARE: integrated pose, angle, and range stabilization**Require:** Image frame $I_{t}$; marker corners $\left\{X_{i}\right\}$; intrinsics K; prior state $\left({\hat{x}}_{t-1},P_{t-1},\kappa_{t-1}\right)$; parameters $\left(\alpha_{z},\eta ,Q,R_{\min},\gamma ,z_{\mathrm{ref}},\tau_{z},\tau_{r}\right)$**Ensure:** Fused angles $\left(\theta_{h}^{\mathrm{fus}},\theta_{v}^{\mathrm{fus}}\right)$; marker-normal heading $\psi_{\mathrm{yaw}}$; fused translation $t^{\mathrm{fused}}$; filtered state $\left({\hat{x}}_{t},P_{t}\right)$  1:**Step 1: Geometric pose recovery**  2:Detect ArUco corners $\left\{x_{i}\right\}$ in $I_{t}$; solve PnP →(r,t)  3:Convert r→R via Rodrigues (Equation (5)); recover marker-normal heading $\psi_{\mathrm{yaw}}$ (Equation (7))  4:**Step 2: Dual-path angular estimation**  5:Image-based: $\left(\alpha_{h},\alpha_{v}\right)$ from Equations (8) and (9)  6:Geometry-based: $\left(\beta_{h},\beta_{v}\right)$ from Equations (10) and (11)  7:**Step 3: MiDaS depth fusion with EMA scale**  8:$D_{\mathrm{rel}}\leftarrow \Phi_{\mathrm{MiDaS}}\left(I_{t}\right)$; compute ${\tilde{d}}_{\mathrm{rel}}$ (Equation (18))  9:Instantaneous alignment ${\hat{\kappa }}_{t}$ (Equation (19)); EMA: $\kappa_{t}$ (Equation (20))10:Fuse: $t_{z}^{\mathrm{fused}}$ (Equation (22)); update $t^{\mathrm{fused}}$ (Equation (23))11:**Step 4: Adaptive angle fusion**12:Compute ρ and $q_{z}$ (Equations (27) and (28)), then weights $w_{\mathrm{img}},w_{\mathrm{tvec}}$ (Equations (29) and (30))13:Fuse: $\left(\theta_{h}^{\mathrm{fus}},\theta_{v}^{\mathrm{fus}}\right)$ (Equations (31) and (32))14:**Step 5: Adaptive Kalman smoothing**15:Predict $\left({\hat{x}}_{t|t-1},P_{t|t-1}\right)$; compute the continuous innovation-dependent covariance $R_{t}$ (Equation (35)); update $\left({\hat{x}}_{t},P_{t}\right)$ and commit the filtered bearing together with the current range output16:**Step 6 (conditional extension): IMU fusion**17:If IMU data are enabled, compute $q_{\mathrm{fused}}\leftarrow \mathrm{SLERP}\left(q_{\mathrm{vis}},q_{\mathrm{imu}},\lambda \right)$ (Equation (40))

In the quadratic branch, the state correction is

(39) $$\Delta x_{t}=\frac{P_{t|t-1}\nu_{t}}{P_{t|t-1}+\gamma \nu_{t}^{2}}.$$

Thus, $|\Delta x_{t}|$ decreases toward zero as $|\nu_{t}|$ becomes very large, giving a bounded-influence robustification of the scalar update. The rule is transparent and inexpensive, but it is heuristic rather than a statistically identified adaptive-covariance estimator. The experiments support it only through a same-pipeline bearing ablation; they do not constitute a comprehensive comparison with covariance-matching, Sage–Husa, or Huberized Kalman filters.

### 3.9. Quaternion-Based IMU Fusion (Optional Extension)

An optional fusion path can augment the visual pipeline under severe sea states. It combines vision-derived and IMU-derived orientations in quaternion space, SO(3), by spherical linear interpolation (SLERP):

(40) $$q_{\mathrm{fused}}\left(\lambda \right)=\frac{\sin\;\left((1-\lambda )\Omega \right)}{\sin\Omega }\;q_{\mathrm{vis}}+\frac{\sin(\lambda \Omega )}{\sin\Omega }\;q_{\mathrm{imu}},$$

where $\Omega =\arccos(q_{\mathrm{vis}}\cdot q_{\mathrm{imu}})$ is the geodesic angle between the two quaternions and λ∈[0,1] balances vision (λ→0) against inertia (λ→1). SLERP guarantees shortest-path interpolation on the unit 3-sphere and avoids gimbal-lock artifacts inherent in Euler-angle blending [36]. When Ω approaches zero, linear interpolation with subsequent normalization is substituted to maintain numerical stability. The default VARE configuration evaluated in the main experiments is vision-only; this IMU path is retained only as an optional implementation extension and is not included in the main quantitative ranking.

### 3.10. Numerical and Practical Considerations

Several implementation details are critical for achieving robust performance in practice:

- Lens distortion correction: All detected corner coordinates are undistorted using the calibrated radial ($k_{1},k_{2}$) and tangential ($p_{1},p_{2}$) distortion parameters prior to PnP computation, eliminating systematic nonlinear perturbations.
- Unit consistency: Projection and fusion equations are evaluated in radians; the final Kalman smoothing and all reported angular metrics use degrees, matching the logging interface and Table 5.
- Marker-frame corner specification: For a square marker of side length *L* with the origin of $F_{M}$ at the marker center and $Z_{M}$ along the board normal, the canonical 3D corners are [−L/2,−L/2,0], [L/2,−L/2,0], [L/2,L/2,0], and [−L/2,L/2,0] in meters.

### 3.11. Algorithm Summary

The complete VARE pipeline is summarized in Algorithm 1.

The default VARE configuration evaluated in the main experiments is vision-only and stops after Step 5. Step 6 remains a conditional extension and is not included in the main quantitative tables.

## 4. Experimental Evaluation

### 4.1. Experimental Configuration

Evaluation was organized into three campaigns. A land test measured angular error under controlled geometry. Pool trials introduced vehicle motion and water-surface disturbance. Near-shore river, harbor, and lake trials examined representative operation in field imagery. Quantitative errors were computed by pairing the VARE overlay/logged measurements with synchronized independent reference values obtained from external surveying instrumentation. In the dynamic water-surface experiments, survey-grade RTK-GNSS measurements provided the time-aligned relative geometry after applying the measured antenna-to-camera and antenna-to-marker lever arms. The ablations used the pool data because that campaign contained repeated approaches under comparable conditions.

#### 4.1.1. Hardware, Software Platform, and Runtime Budget

The Python 3.12 implementation used OpenCV 4.5 [35], PyTorch 1.12 and the MiDaS DPT_Large backbone. It ran on an NVIDIA Jetson AGX Xavier in 30 W mode (Santa Clara, CA, USA). MiDaS inference used TensorRT FP16, and the processed camera stream was 1920×1080 at 30 fps.

Table 3 reports the archived runtime measurements. MiDaS runs asynchronously at approximately 10 Hz, while ArUco detection, PnP, bearing fusion, and filtering follow the camera stream. The latest completed MiDaS cue is read without blocking the synchronous stage. We distinguish (i) fused-output throughput, (ii) per-inference computation time, and (iii) measurement data age. For a MiDaS input captured at $t_{\mathrm{capture}}$ and consumed by fusion at $t_{\mathrm{use}}$, the data age is $A_{t}=t_{\mathrm{use}}-t_{\mathrm{capture}}$. The archived profiler contains aggregate computation-time and update-rate summaries but not the paired frame-wise capture and use timestamps required to report the distribution of $A_{t}$.

**Figure 2:**
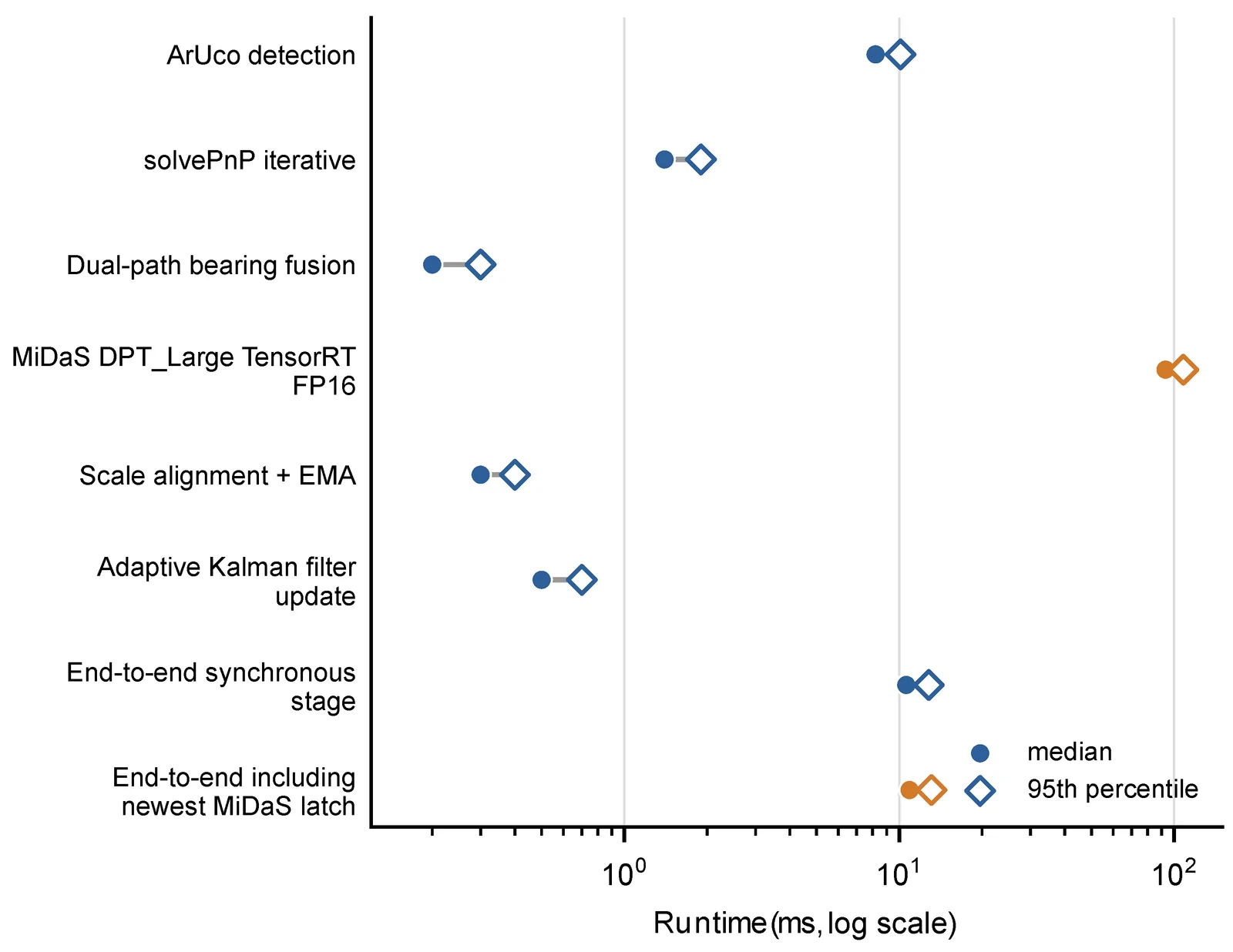
Per-module runtime on the Jetson AGX Xavier. Blue symbols represent original pipeline stages. Orange/yellow symbols denote components involving the heavy MiDaS DPT_Large FP16 depth-estimator. Points and whiskers are computed directly from Table 3; circles show median computation time and diamonds show the 95th percentile. The MiDaS branch is asynchronous, while the CPU-only synchronous stage remains near the camera-frame rate.

MiDaS dominates the reported computation time: one inference takes 93 ms at the median and 108 ms at the 95th percentile, whereas the fused output is produced at 28–30 Hz by reusing the latest completed cue Figure 2. The latter is output throughput, not a 28–30 Hz fresh-depth rate and not a bound on $A_{t}$. A lighter depth backbone could reduce this imbalance, but it was not evaluated here.

#### 4.1.2. Sensor and Marker Configuration

**Figure 3:**
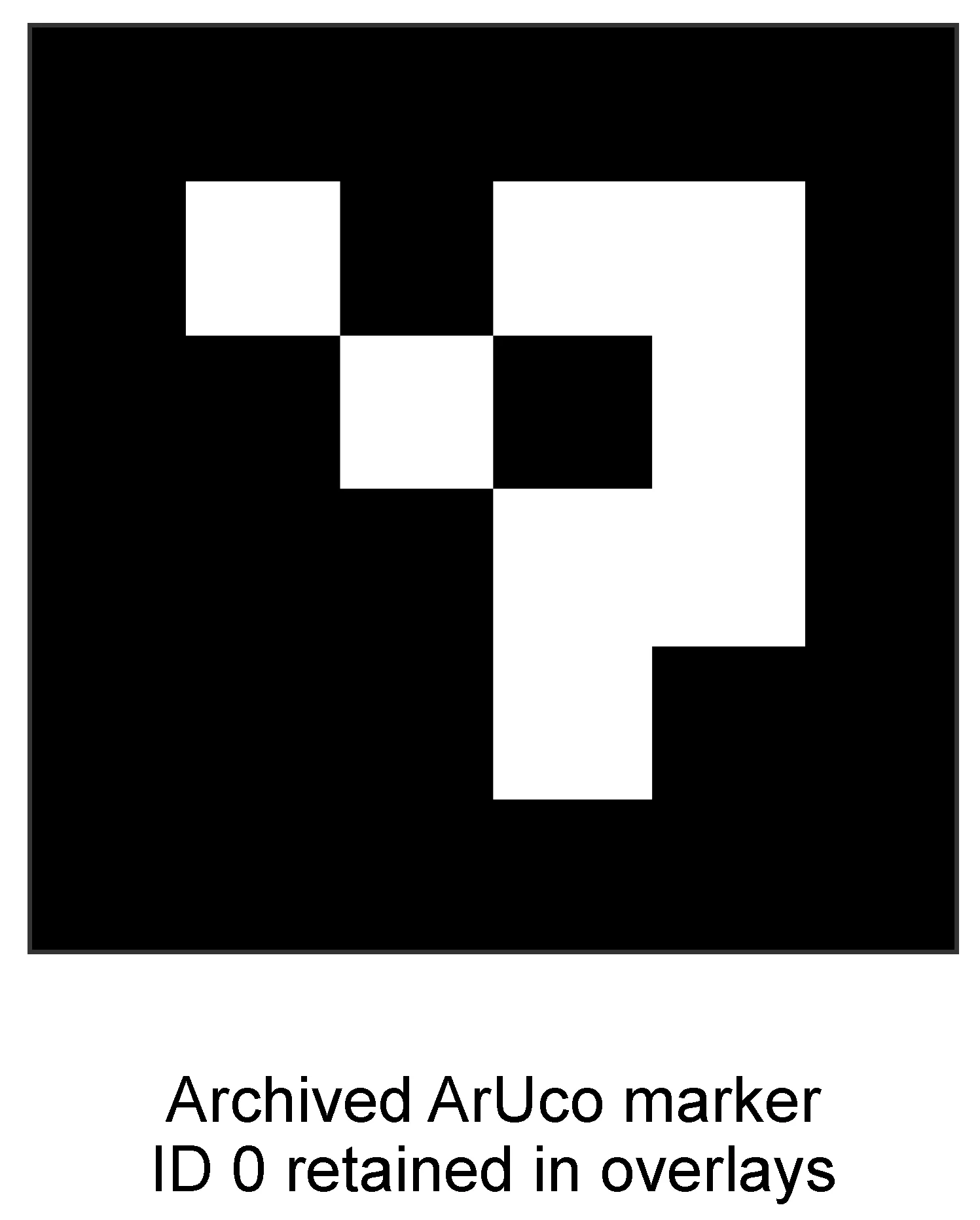
ArUco marker pattern used in the archived experiments. The marker structure is preserved, and the field overlays identify the detected marker as ID 0.

A calibrated monocular camera was mounted on the USV bow Figure 3. The processed stream was 1920×1080 at 30 fps. Project notes describe a horizontal field of view of 58–60°, which corresponds to an effective horizontal focal length of approximately $\left(1.66–1.73\right)\times 10^{3}$ pixels for the processed stream. The square marker side length was 500 mm. Every field overlay identifies the detected marker as ID 0, and the figure captions follow the visible overlay evidence. The exact camera intrinsic matrix, distortion coefficients, ArUco dictionary and detector settings, acquisition settings, marker specification, and camera/RTK/marker calibration and lever-arm records are available from the corresponding author at chenchen_123@stu.xidian.edu.cn upon reasonable request.

As a long-range visibility sanity check, the archived river overlay at $d_{xz}=70.64$ m corresponds to an expected marker side length of approximately 12 pixels under the reported 58–60° processed-stream field of view. The two closer river overlays at 40–44 m correspond to expected marker side lengths of roughly 19–22 pixels. These marker footprints are small but remain within the operating range of OpenCV’s ArUco detector when the marker is high-contrast and corner refinement is enabled. Table 4 summarizes the camera–marker evidence available from the logged experiments. Because the archived logs do not contain a complete frame-level detection-survival record, the field examples should be interpreted as representative detections rather than as a full dropout-rate benchmark.

#### 4.1.3. Hyperparameter Settings

The complete set of hyperparameters used in all experiments is listed in Table 5. These values were determined through preliminary calibration runs on the land dataset and then held fixed across all subsequent experiments (pool and water surface). They should therefore be interpreted as reasonable global operating settings rather than scenario-wise optima.

The Kalman parameters are expressed in deg^2^ because the final smoothing stage operates on the logged angular outputs after conversion from radians. The filter is updated at the 30 fps camera rate, while the asynchronous MiDaS scale update is latched at approximately 10 Hz, as described in Table 3. The optional IMU coefficient is omitted from Table 5 because no IMU-assisted configuration is included in the quantitative evaluation.

#### 4.1.4. RTK-Synchronized External Reference Construction

Pool and near-shore water-surface experiments used independent RTK-GNSS reference records synchronized with the camera timeline. The camera timestamp $t_{c}$ was matched to the nearest RTK epoch $t_{r}$ after clock-offset calibration; when the residual offset exceeded one RTK sampling period, linear interpolation between adjacent RTK poses was used. The RTK antenna position on the USV, the camera optical center, and the marker reference point are not collocated. Therefore, measured lever arms were applied before constructing reference bearing and range.

Let $p_{A}^{W}\left(t\right)$ denote the RTK antenna position of the camera platform in the world frame, $R_{B}^{W}\left(t\right)$ the body-to-world attitude used for lever-arm compensation, and $\ell_{AC}^{B}$ the measured vector from the RTK antenna to the camera optical center in the body frame. The required attitude terms were obtained from the platform heading log and the surveyed camera/marker mounting orientation; for fixed infrastructure markers, the marker orientation was obtained from the surveyed static board installation. The reference camera position is

(41) $$p_{C}^{W}\left(t\right)=p_{A}^{W}\left(t\right)+R_{B}^{W}\left(t\right)\ell_{AC}^{B}.$$

Similarly, the marker-center reference point is obtained from the marker-side RTK/reference point $p_{M,A}^{W}\left(t\right)$ and the measured marker lever arm $\ell_{AM}^{B}$:

(42) $$p_{M}^{W}\left(t\right)=p_{M,A}^{W}\left(t\right)+R_{M}^{W}\left(t\right)\ell_{AM}^{B}.$$

The relative vector is then transformed into the camera frame using the calibrated camera extrinsics,

(43) $$\Delta^{C}\left(t\right)=R_{W}^{C}\left(t\right)\left(p_{M}^{W}\left(t\right)-p_{C}^{W}\left(t\right)\right),$$

from which the independent reference bearing and camera-frame horizontal approach range are computed as

(44) $$\begin{array}{cc} \theta_{h}^{\mathrm{ref}}\left(t\right) & =atan2(\Delta_{x}^{C}\left(t\right),\Delta_{z}^{C}\left(t\right)), \end{array}$$

(45) $$\begin{array}{cc} d_{xz}^{\mathrm{ref}}\left(t\right) & =\sqrt{{\left(\Delta_{x}^{C}\left(t\right)\right)}^{2}+{\left(\Delta_{z}^{C}\left(t\right)\right)}^{2}}. \end{array}$$

Because the bow camera was mounted approximately level during the evaluated approach windows, the camera-frame $X_{C}$–$Z_{C}$ range is used as the horizontal approach-range metric. Equations (41)–(45) ensure that the reference values are external to VARE. They are not reconstructed from VARE outputs and are not generated by post hoc fitted error models.

#### 4.1.5. Reference Values and Error Metrics

The experiments used independent external reference measurements rather than references reconstructed from VARE outputs. Land tests used calibrated fixture distances, lateral offsets, and board orientations to define the angular reference. Pool and near-shore water-surface tests used the RTK-synchronized reference construction in Section 4.1.4; the measured antenna-to-camera and antenna-to-marker lever arms were applied before computing the reference line of sight and camera-frame horizontal approach range.

For each sampled frame or steady evaluation window, the reference bearing and range were computed from the independently measured relative geometry, while the measured values were taken from the VARE output logs or archived overlay readouts. Angular error is reported as RMSE in degrees. Range error is reported as MAE or RMSE in meters using $e_{d}=d_{xz}^{\mathrm{meas}}-d_{xz}^{\mathrm{ref}}$. Bearing error is computed as $e_{\theta }=\theta_{h}^{\mathrm{meas}}-\theta_{h}^{\mathrm{ref}}$ for the horizontal channel and analogously for the vertical channel when available. Trial-level statistics are computed by first evaluating each approach independently and then summarizing the distribution across trials. The reported values are experimental summaries derived from independent references; they are not generated from distance-dependent formulas fitted to the measured outputs.

#### 4.1.6. Evidence Release and Interpretation Boundary

The materials available for this study comprise the manuscript figures, table-level summaries, representative overlay frames, synchronized RTK/reference summaries, lever-arm and timestamp-matching records, trial-level metric summaries, aggregate runtime summaries, and the camera/marker calibration assets listed in Section 4.1. These materials are available from the corresponding author at chenchen_123@stu.xidian.edu.cn upon reasonable request. A public frame-wise detection log and evaluation-code archive is not presently available; accordingly, the paper does not report an exact detection-survival curve, marker-dropout rate, MiDaS data-age distribution, or closed-loop recovery probability. The reported percentage reductions are descriptive experimental comparisons under the documented reference construction rather than claims of universal performance.

#### 4.1.7. Comparison Methods and Ablation Variants

VARE was compared against one pixel-only monocular baseline, one strong planar-marker geometry baseline, and two reduced variants derived from the proposed pipeline:

- Pixel-only baseline (monocular): Horizontal and vertical bearing are computed directly from image-plane centroid offsets, and range is estimated from apparent marker size using the pinhole camera model. This baseline does not use solvePnP, MiDaS depth, confidence-weighted fusion, or innovation-adaptive filtering. Accordingly, it provides image-plane bearing and monocular range only; it does not recover a 3D pose and therefore does not define the marker-normal heading variable in the same sense as the pose-capable variants below.
- Variant A (PnP + static KF): Standard ArUco detection with solvePnP (iterative solver), followed by a conventional Kalman filter with fixed measurement noise R=0.5 deg^2^.
- Variant B (PnP + direct MiDaS substitution): The PnP vector is uniformly rescaled as $t^{B}=\left(d_{\mathrm{midas}}/t_{z}\right)t$, with static filtering and no adaptive depth fusion. The rescaling itself changes range magnitude while preserving the raw translation-vector direction. In the complete evaluation pipeline, however, the substituted depth is also passed to the depth-dependent confidence module, so bearing metrics may differ from Variant A through weighting rather than through a rotation of the PnP vector.
- IPPE-square + One-Euro baseline: The square-marker pose is estimated with OpenCV SOLVEPNP_IPPE_SQUARE. The image-corner order follows the same ArUco detection order as the center-origin object points in Equation (3), and the same camera intrinsics, distortion model, frame set, RTK/reference values, and evaluation script are used as for VARE. The resulting translation and bearing channels are smoothed with a One-Euro filter using the same parameter family as the other smoothing controls. This baseline is included as a stronger geometry reference because IPPE is designed for planar targets and the One-Euro filter is an effective lightweight smoother for interactive pose streams.

The pixel-only baseline was recomputed offline from the same recorded image observations and evaluated against the same independent references; it was not executed as a separate onboard controller stack. Variants A and B are internal controls, whereas the IPPE-square + One-Euro row provides a matched geometry-oriented reference for planar-marker pose estimation. Table 6 separates matched quantitative comparisons from contemporary system classes whose required inputs or outputs are absent from the retained archive. Numerical performance for the latter systems is not reported, and the manuscript does not claim state-of-the-art superiority over full docking, AprilTag-board, metric-depth, learning-refinement, or visual–inertial systems.

### 4.2. Experiment 1: Controlled Land Test

#### 4.2.1. Objective and Setup

The land test isolated angular behavior from hydrodynamic disturbance. A tripod-mounted camera observed two 500 mm marker boards at 10, 20, 30, and 40 m. The two boards used the same printed pattern and were analyzed separately for repeatability.

#### 4.2.2. Procedure

**Figure 4:**
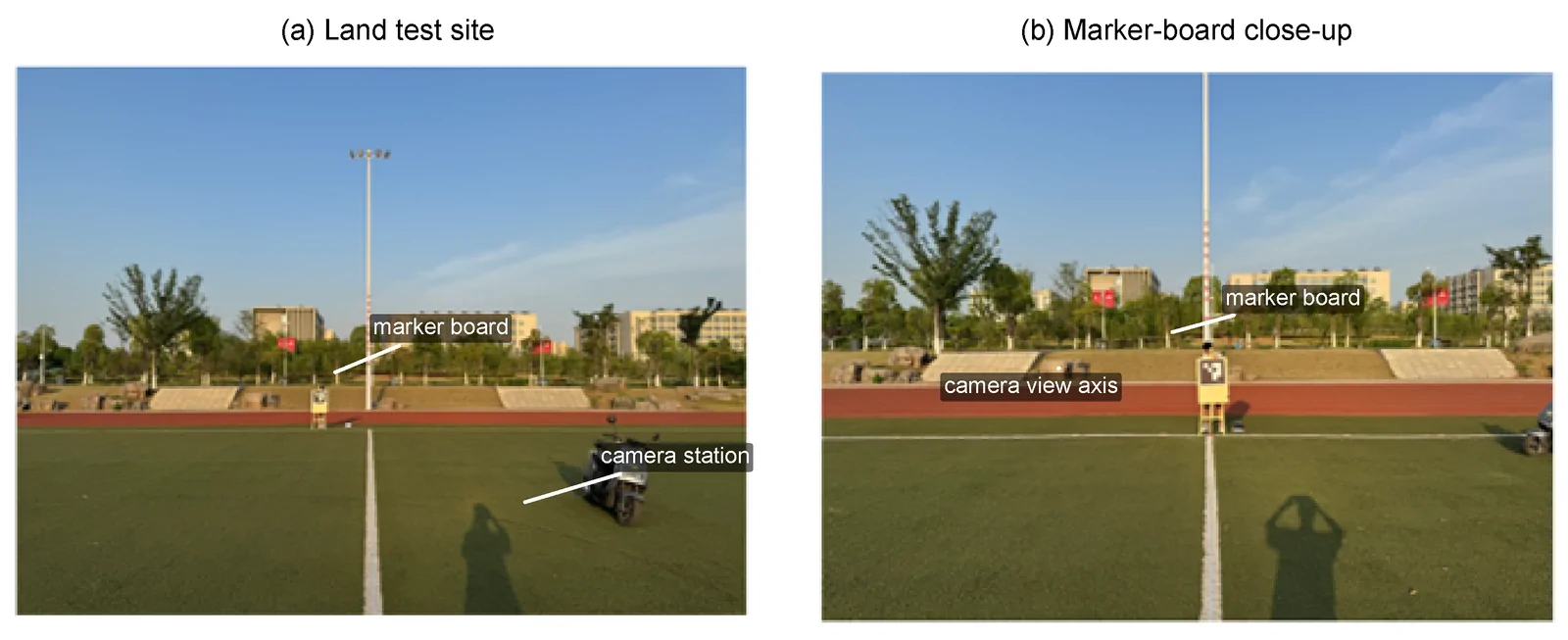
Controlled land experiment environment. (**a**) Open-field test site with the camera station and calibrated marker board. (**b**) Close-up view of the marker-board setup. Labels indicate only equipment visible in the source image.

At each distance, the camera was displaced laterally in calibrated increments (0.5 m at 10 m range, increasing to 1.5 m at 40 m range) to generate varying horizontal angles Figure 4. At each lateral position, six angular orientations spanning −30° to +30° were tested, producing between 10 and 15 independent measurement configurations per distance.

#### 4.2.3. Results

**Figure 5:**
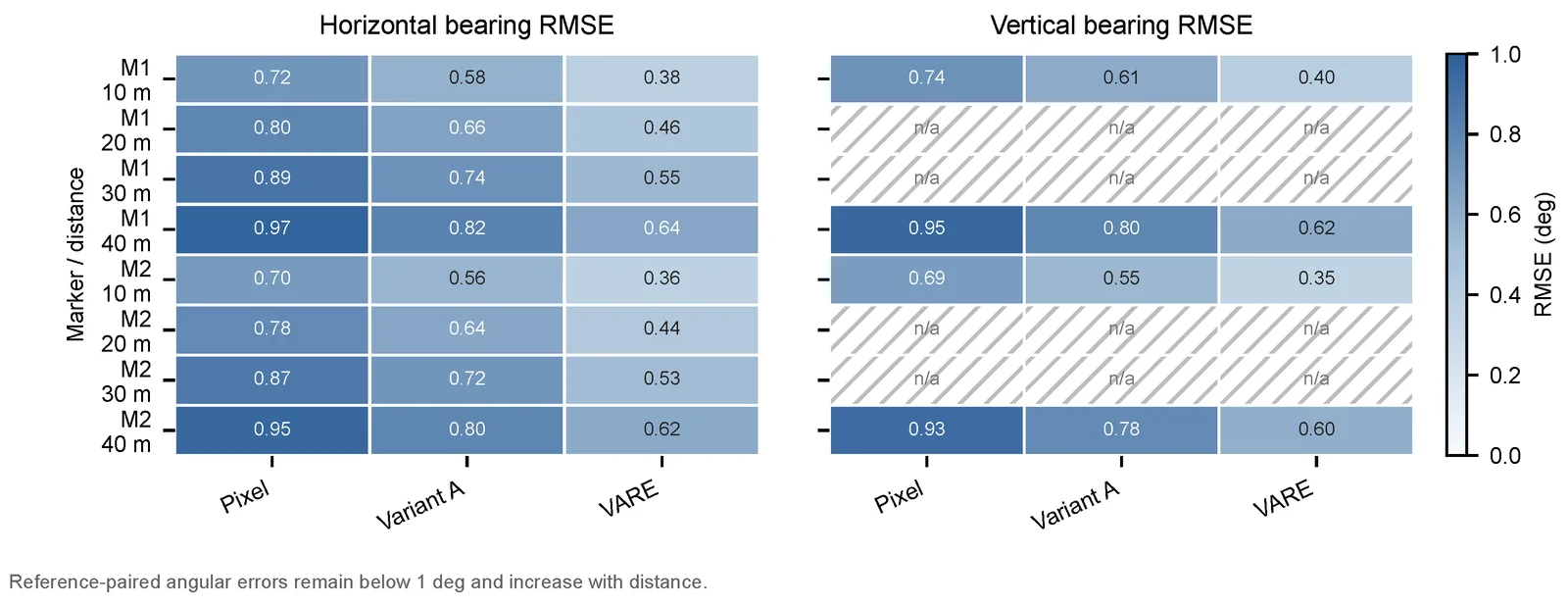
Land-test angular RMSE summarized from Table 7. Separate horizontal and vertical panels avoid mixing axes. Hatched cells mark distances where the table reports a single value without an explicit separate vertical RMSE entry.

The quantitative results are presented in Table 7 and Figure 5. To enable direct comparison, both the pixel-only baseline and Variant A (PnP + static KF) were evaluated on the same data. Variant B is omitted here because the land experiment is primarily designed to stress the angular channel rather than the depth-stabilization branch.

#### 4.2.4. Interpretation

VARE has the lowest independent-reference angular RMSE in every land-test row. Across all reported horizontal and vertical entries, the reduction is 34.0–49.3% relative to the pixel-only baseline and 22.0–36.4% relative to Variant A. Both boards show the same method ordering. The absolute error increases with distance for each reported method, which is consistent with the expected effect of decreasing marker image footprint and increasing sensitivity to small corner-localization errors. The dataset is still too small to separate the effects of range weighting, calibration, corner localization, and viewing angle. The result therefore supports the dual-path design under the tested configurations without establishing a general distance law.

### 4.3. Experiment 2: Pool-Based Model Test

#### 4.3.1. Objective and Setup

**Figure 6:**
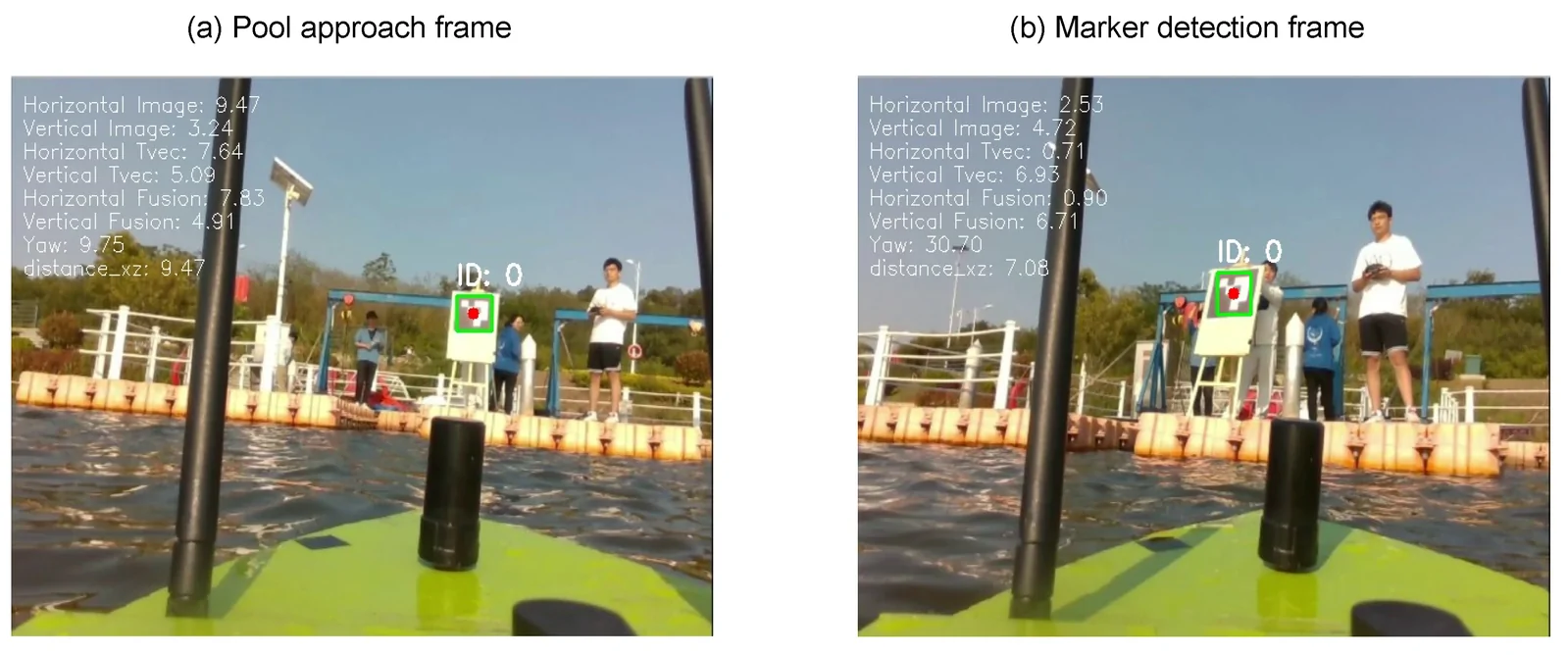
Pool-based model experiment. (**a**,**b**) Representative onboard-camera frames during approach maneuvers. The original overlay detections and ID 0 marker display are retained; added labels identify visible platform and marker context only.

The pool test introduced wake, reflection, and propulsion vibration while preserving repeatable approach geometry. A scaled USV approached a fixed marker at the pool edge Figure 6. Synchronized RTK reference summaries and measured setup geometry were used to compute the reference bearing and range for the evaluated steady windows. The present archive preserves the reported summaries but not a public frame-wise evaluation script or detection-status log.

#### 4.3.2. Procedure

Fifteen approaches covered approximately 2–10 m. Each trial lasted about 30 s at 30 fps, corresponding to a nominal acquisition scale of approximately 13,500 frames across the campaign. Because “about 30 s” is a rounded duration and the frame-wise detection log is not preserved, 13,500 is not reported as an exact evaluable- or detected-frame count. Horizontal bearing RMSE was computed against the synchronized external reference. Range MAE and range RMSE were computed by comparing the VARE range output with the corresponding RTK-derived reference range at the evaluated timestamps. These metrics quantify meter-scale absolute range error rather than frame-to-frame variation.

#### 4.3.3. Results

The quantitative results for the pool experiment are summarized in Table 8, comparing VARE against the pixel-only baseline, two internal ablation variants, and the IPPE-square strong geometry baseline. In this experiment, the angular metric is horizontal bearing error because the pool setup provides a calibrated visual reference angle but not an independent sensor for validating marker-normal heading.

Using the reported VARE mean Table 9, standard deviation, and N=15, the two-sided 95% confidence intervals of the trial-level mean are [0.42, 0.50] deg for bearing RMSE, [0.75, 0.89] m for range MAE, and [0.82, 0.98] m for range RMSE. These intervals describe uncertainty around each VARE mean; they are not confidence intervals for paired method differences. VARE remains the lowest-error method in the available trial-level summary. The improvement over the IPPE-square + One-Euro baseline is smaller than the improvement over the pixel-only and static-PnP references. The result is therefore interpreted as a targeted descriptive improvement under the recorded pool conditions rather than as a universal dominance or significance claim.

#### 4.3.4. Interpretation

VARE records the lowest vision-only value in all three pool columns. Relative to the pixel-only baseline, bearing RMSE decreases by 47.7%, range MAE by 38.8%, and range RMSE by 38.8%. Relative to Variant A, the corresponding reductions are 35.2%, 26.8%, and 26.8%. Relative to the stronger IPPE-square + One-Euro baseline, VARE reduces bearing RMSE by 14.8%, range MAE by 4.7%, and range RMSE by 5.3%.

The range metrics compare overlay/logged measured range with RTK-derived reference range. They therefore describe meter-scale range error rather than frame-to-frame smoothness alone. The adaptive Kalman state is angular and has no binary range-commit gate; range performance is attributed to the geometry/MiDaS range branch, not to the scalar bearing filter.

### 4.4. Experiment 3: Near-Shore Water Surface Trial

#### 4.4.1. Objective and Setup

The field trial examined VARE under reported real-world maritime conditions including ambient wave motion (Sea State 1–2), solar glare, wind-driven ripples, and varying illumination. These observations cover mild-to-moderate conditions; they do not constitute controlled tests of severe backlighting, heavy rain or spray, saturated reflection, or rapidly changing high sea states. Three field scenarios were used to examine complementary aspects of the framework:

- Scenario A: River navigation approach. The camera system was mounted on the bow of a USV prototype navigating a river channel toward a navigation beacon (red tower) with the ArUco marker board affixed to its base. This scenario provides selected detections during an approach from approximately 70 m to 17 m while the USV undergoes natural course corrections; it is not used to infer uninterrupted tracking or a detection rate.
- Scenario B: Harbor dock approach. The USV approached a concrete quay wall in a harbor basin, with the marker mounted on the dock infrastructure near a gantry crane. This scenario adds cluttered visual backgrounds, nearby metallic structures with potential reflection artifacts, and the confined maneuvering space typical of docking.
- Scenario C: Lake platform approach. Repeated open-lake approaches were carried out toward a floating platform equipped with an ArUco marker board. This scenario emphasizes oblique approach geometries under wave disturbance, with sampled frames spanning approximately 4.7–17.5 m in horizontal distance.

In the field scenarios, survey-grade RTK-GNSS logs provide synchronized external reference geometry. The quantitative tables retain the on-screen overlay readouts as the experimental measurements and pair them with RTK-derived reference values. The overlay displays six diagnostic quantities: Horizontal Image ($\alpha_{h}$), Vertical Image ($\alpha_{v}$), Horizontal Tvec ($\beta_{h}$), Vertical Tvec ($\beta_{v}$), Horizontal Fusion ($\theta_{h}^{\mathrm{fus}}$), and Vertical Fusion ($\theta_{v}^{\mathrm{fus}}$). It also displays the marker-normal heading variable ($\psi_{\mathrm{yaw}}$) and camera-frame horizontal approach distance ($d_{xz}$). These readouts provide a direct visual record of dual-path fusion during live operation. The formal docking-alignment criterion used in this paper is Equation (26), namely the simultaneous convergence of fused bearing, marker-normal heading, and lateral offset.

#### 4.4.2. Scenario A: River Navigation Approach Sequence

**Figure 7:**
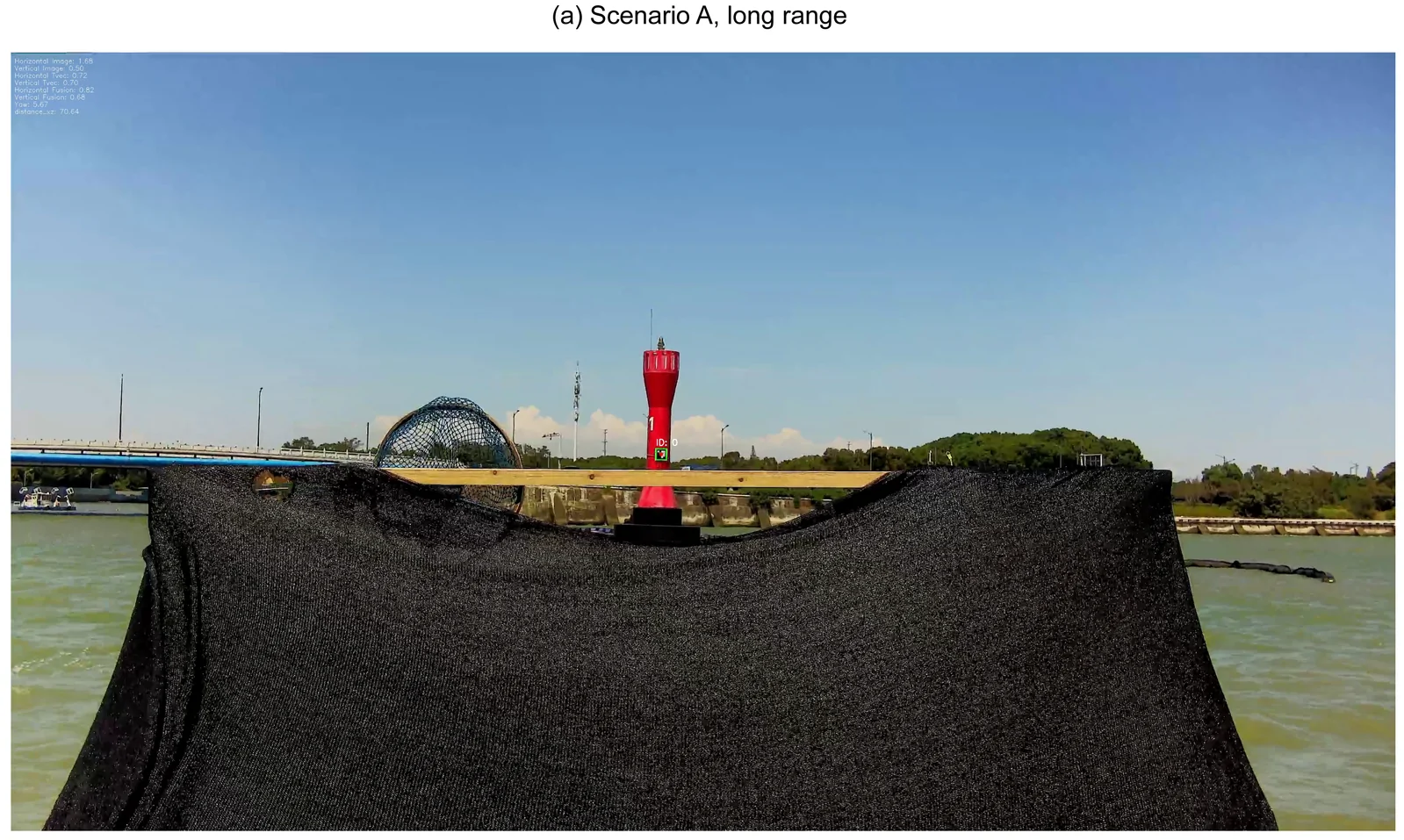
Scenario A archived river-navigation overlay A at long range. The original detection overlay and numeric readouts are retained without modification.

**Figure 8:**
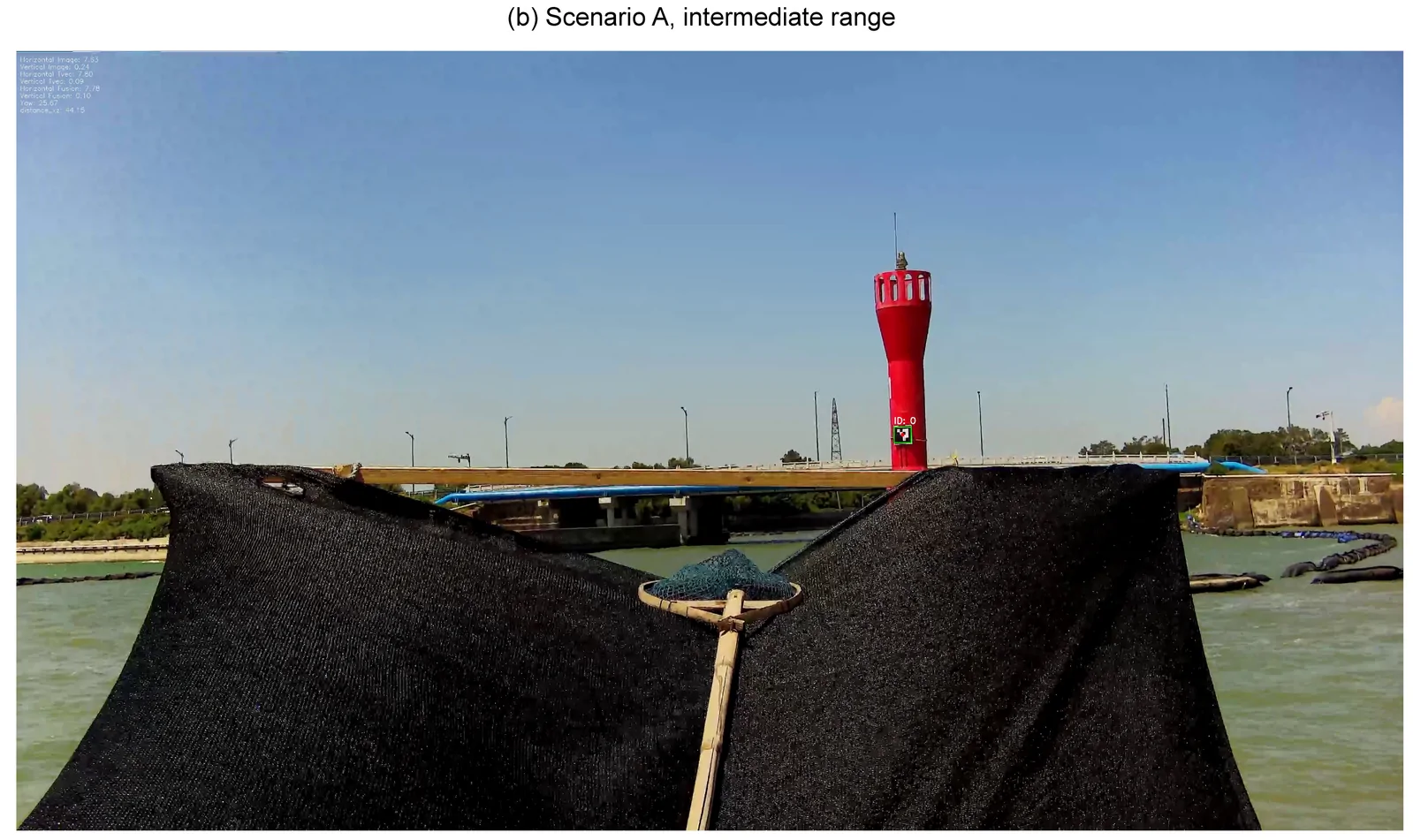
Scenario A archived river-navigation overlay B at intermediate range. The original detection overlay and numeric readouts are retained without modification.

**Figure 9:**
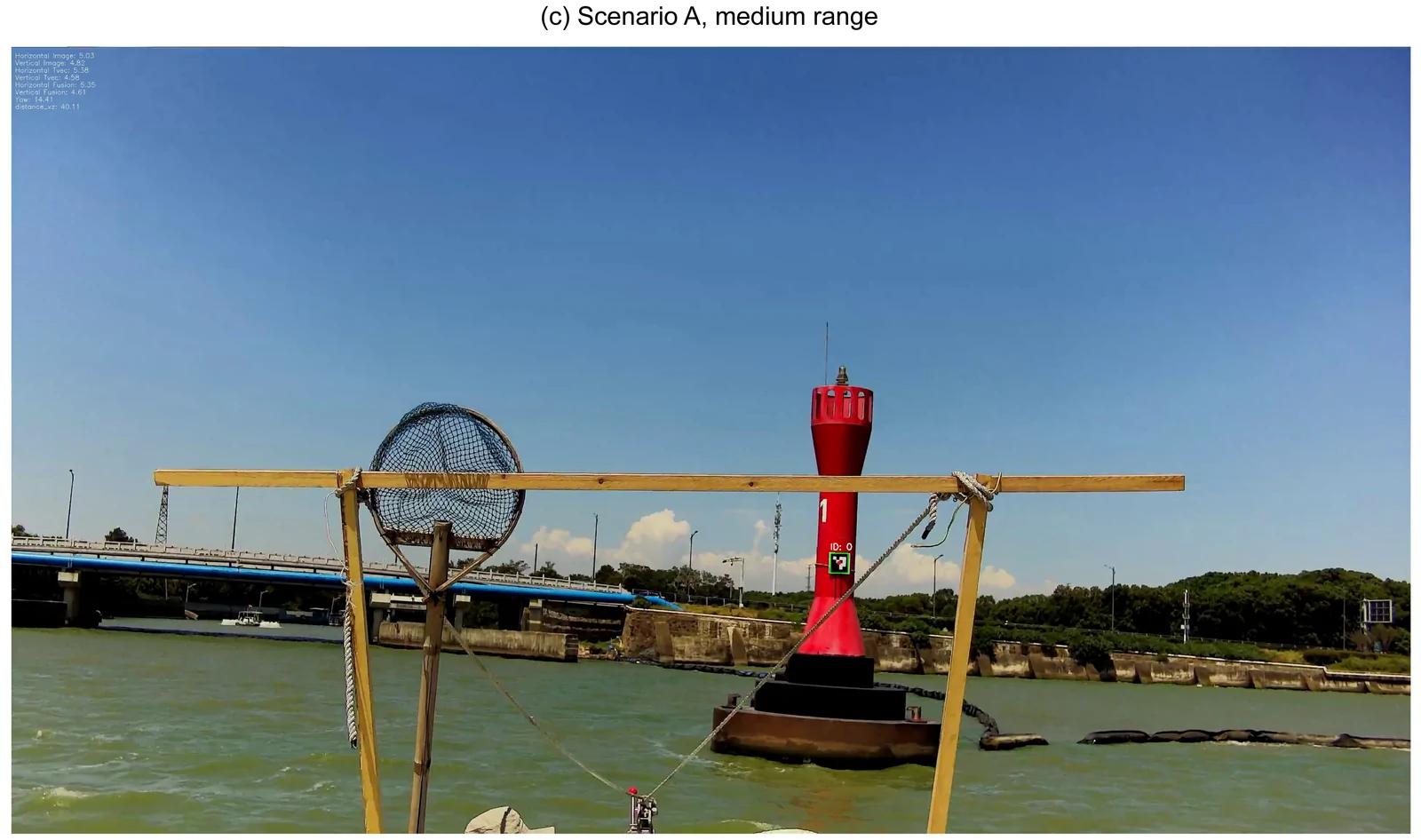
Scenario A archived river-navigation overlay C at medium range. The original detection overlay and numeric readouts are retained without modification.

Figure 7, Figure 8 and Figure 9 show three representative standalone frames from Scenario A, displayed individually at full width. Together, they span a long-range near-centered state, a more oblique mid-range state, and a medium-range state with non-negligible vertical offset. The captions report the overlay values exactly as shown in the recorded frames.

**Figure 10:**
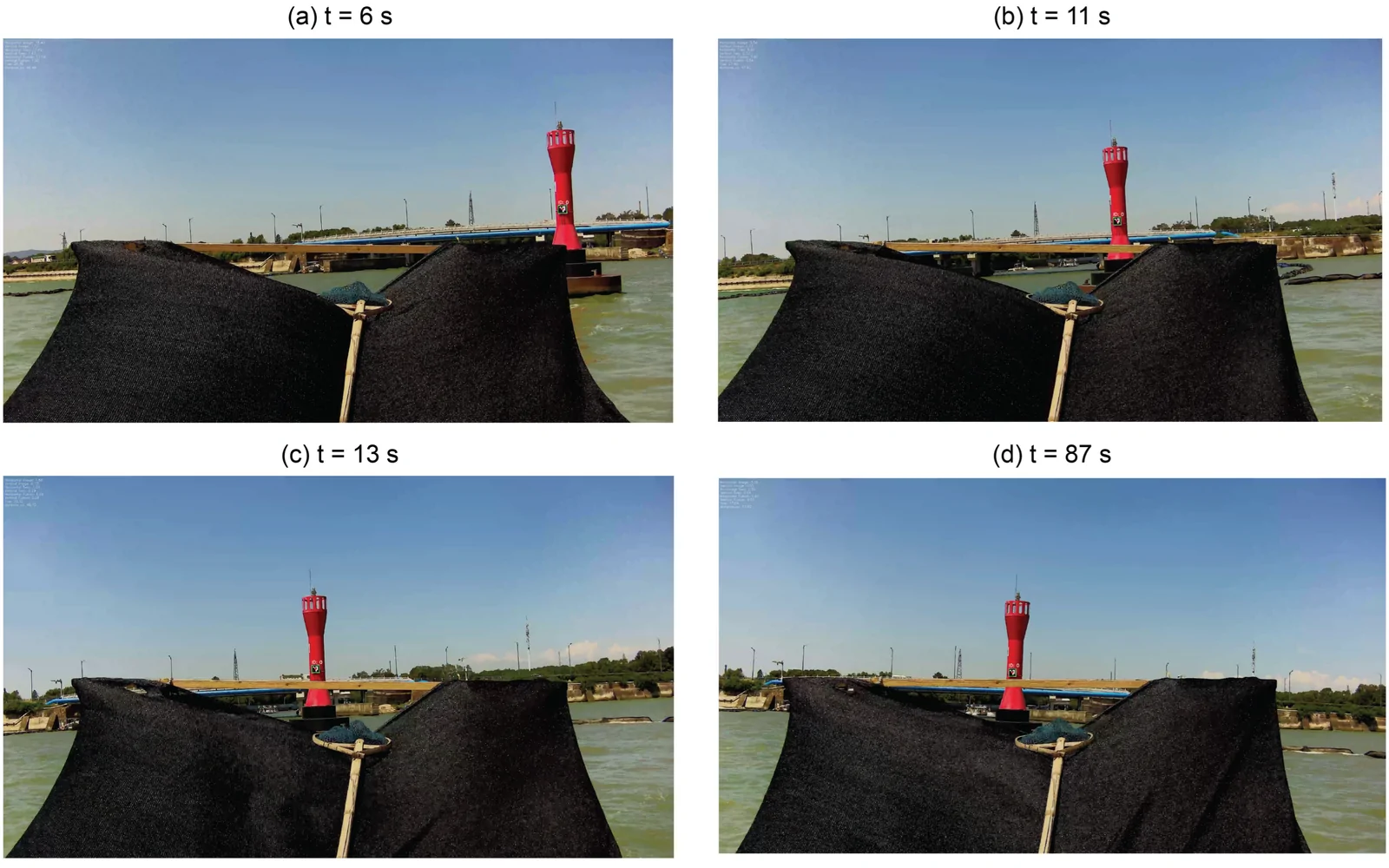
Archived overlay frames from a representative Scenario A open-loop observation sequence. Panel titles give the sampled time stamps; all overlay values and detection marks are retained from the original frames. These frames illustrate open-loop visual perception outputs and should not be read as a closed-loop docking validation.

To demonstrate the temporal dynamics of the alignment process, Figure 10 presents a four-frame time-lapse from a single continuous approach trial in Scenario A. The corresponding quantitative measurements are tabulated in Table 10.

**Figure 11:**
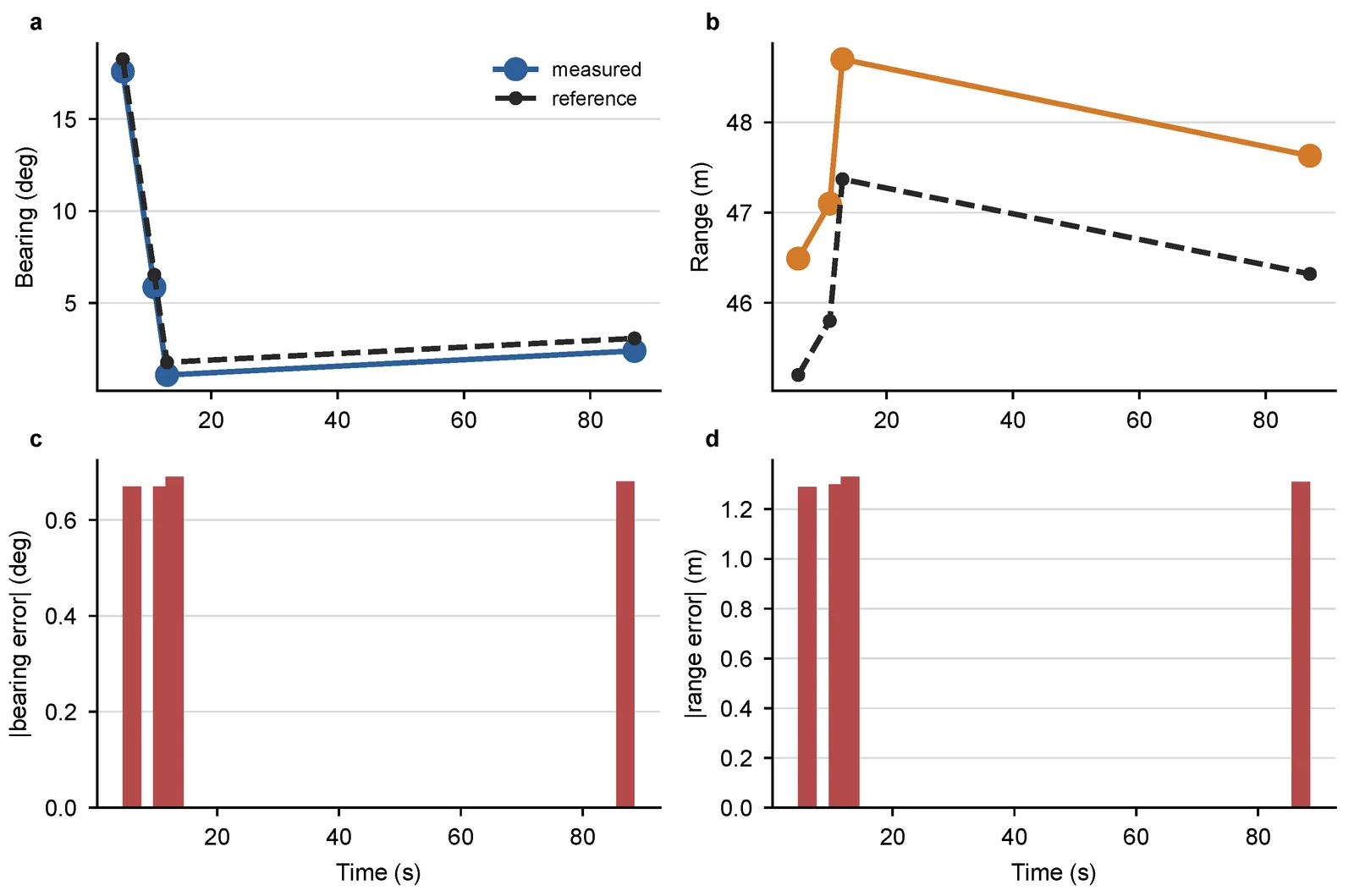
Scenario A measured overlay outputs and RTK-derived reference values computed directly from Table 10. The panels (**a**–**d**) separate measured and reference bearing, measured and reference range, and their absolute errors.

Table 10 and Figure 11 provides an operational diagnostic insight: fused horizontal bearing $\theta_{h}^{\mathrm{fus}}$ can satisfy a visual-centering tolerance before marker-normal heading $\psi_{\mathrm{yaw}}$ has converged. At t=13 s, $\theta_{h}^{\mathrm{fus}}$ has reduced to 1.09° (inside the retrospective tolerance band $|\theta_{h}^{\mathrm{fus}}|<3{}^{\circ}$), while $\psi_{\mathrm{yaw}}$ remains at 20.12°. Under Equation (26), this frame is therefore visually centered but not fully aligned.

Because the formal docking-alignment criterion in Equation (26) includes $\theta_{h}^{\mathrm{fus}}$, $\psi_{\mathrm{yaw}}$, and $t_{x}^{\mathrm{fused}}$, the sequence should be interpreted as evidence of bearing–heading decoupling rather than as a complete docking decision record. A downstream controller may need to regulate visual centering, marker-normal heading, and lateral translation on different time scales.

#### 4.4.3. Scenario B: Harbor Dock Approach

**Figure 12:**
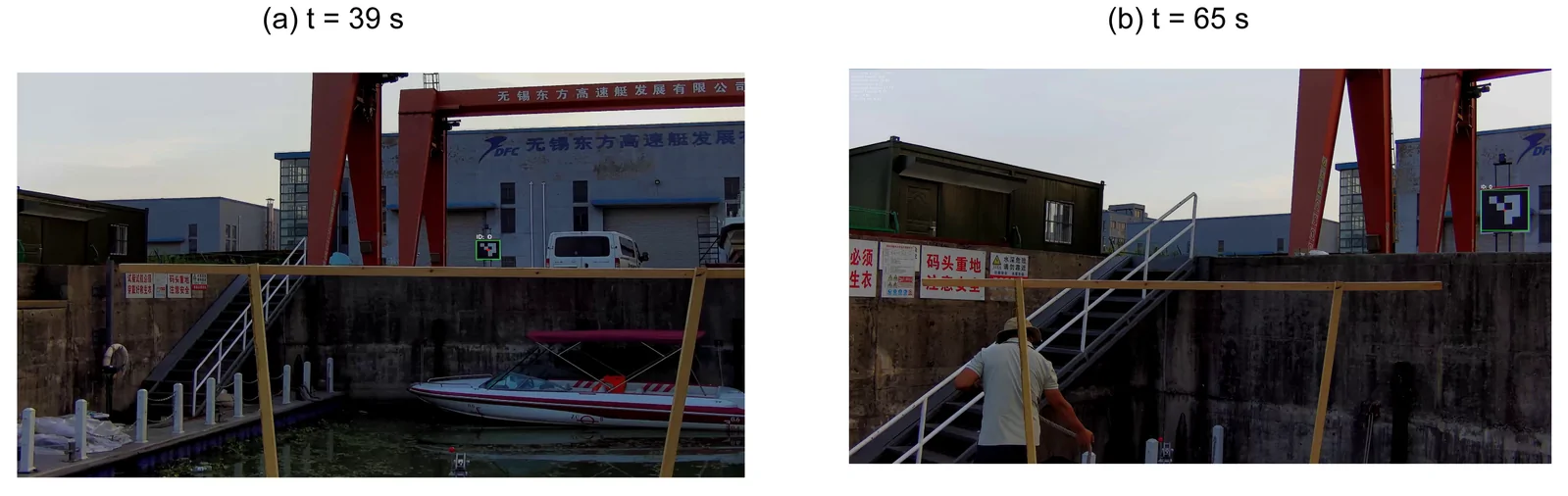
Archived overlay frames from Scenario B near a harbor structure. Panel titles give the sampled time stamps; all overlay values and detection marks are retained from the original frames. The panels show perception outputs under cluttered imagery rather than a full docking benchmark.

Figure 12 presents two representative frames from the harbor dock scenario, illustrating VARE’s operation in a cluttered industrial environment with complex visual backgrounds.

The two harbor frames show successful marker detection against cluttered industrial backgrounds. At 39 s, the bearing is 0.64°, but the corrected overlay value gives a marker-normal heading of 11.38°. This frame is visually centered but not fully aligned under Equation (26). Two selected frames cannot quantify harbor detection robustness or dropout probability.

#### 4.4.4. Scenario C: Lake Platform Approach Trials and Approach Geometry

**Figure 13:**
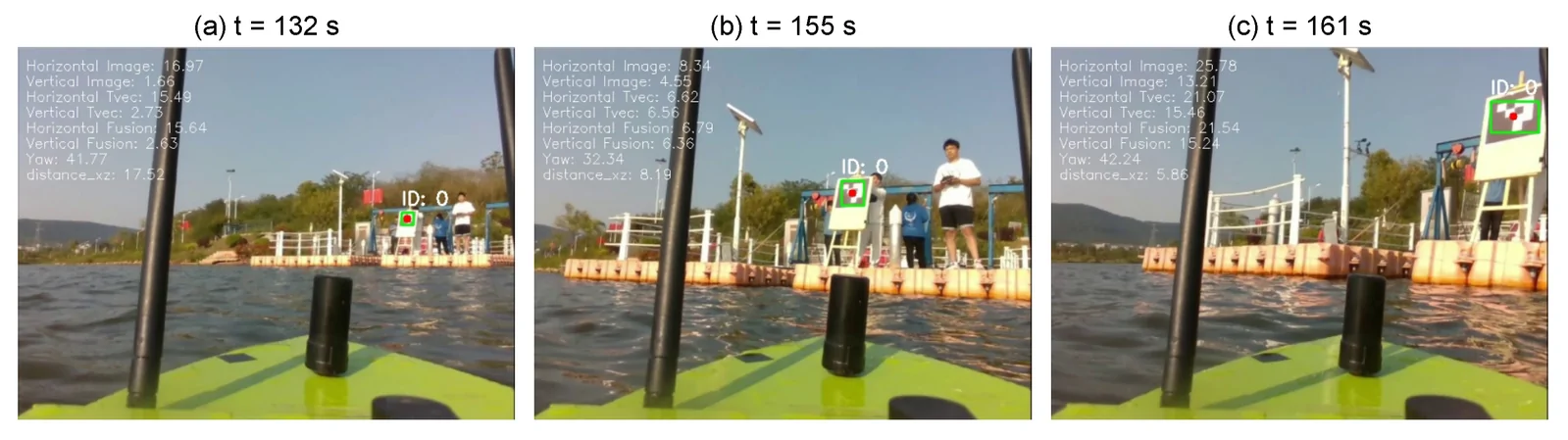
Archived overlay frames from Scenario C Trial 1. The selected frames show persistent large bearing and marker-normal-heading errors during closing; captions report the archived overlay values.

**Figure 14:**
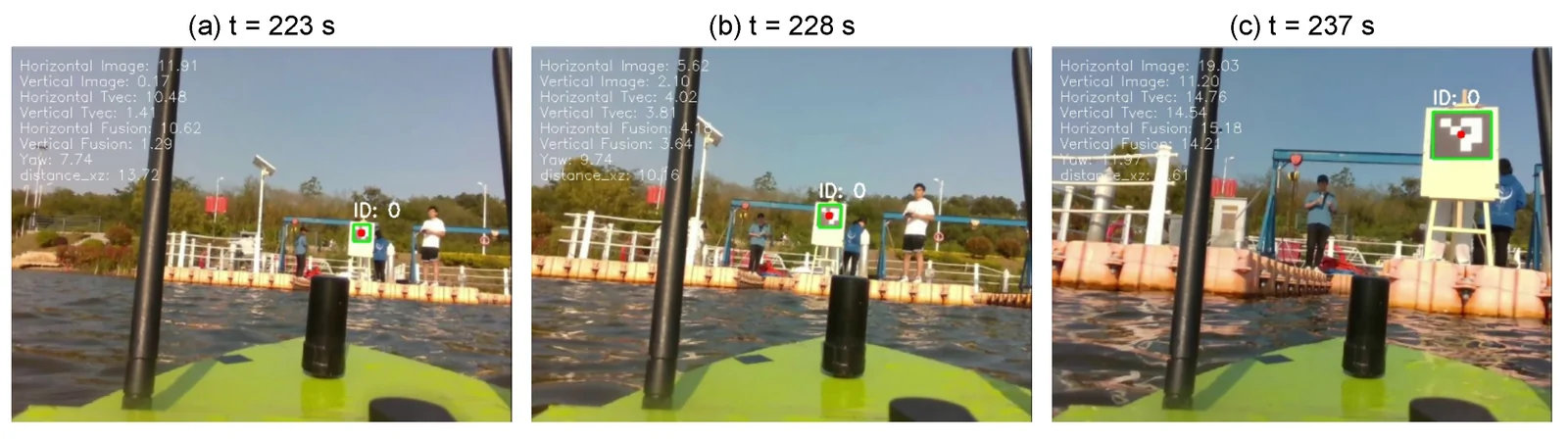
Archived overlay frames from Scenario C Trial 2. The selected frames show partial bearing centering with residual marker-normal-heading error; captions report the archived overlay values.

**Figure 15:**
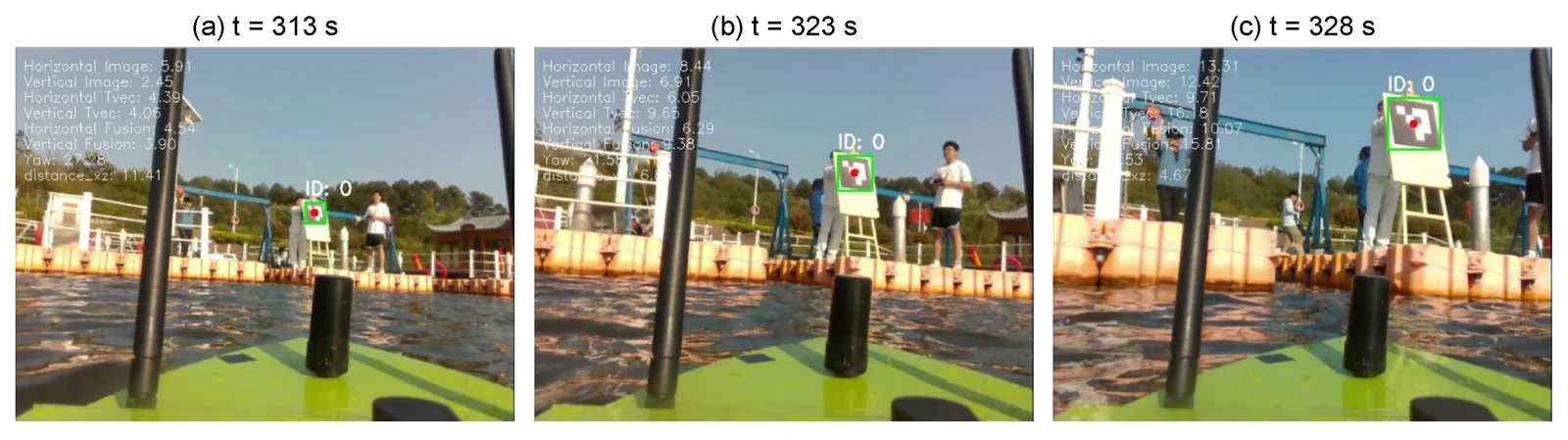
Archived overlay frames from Scenario C Trial 3. The selected frames show moderate bearing with persistent marker-normal-heading error during closing; captions report the archived overlay values.

**Figure 16:**
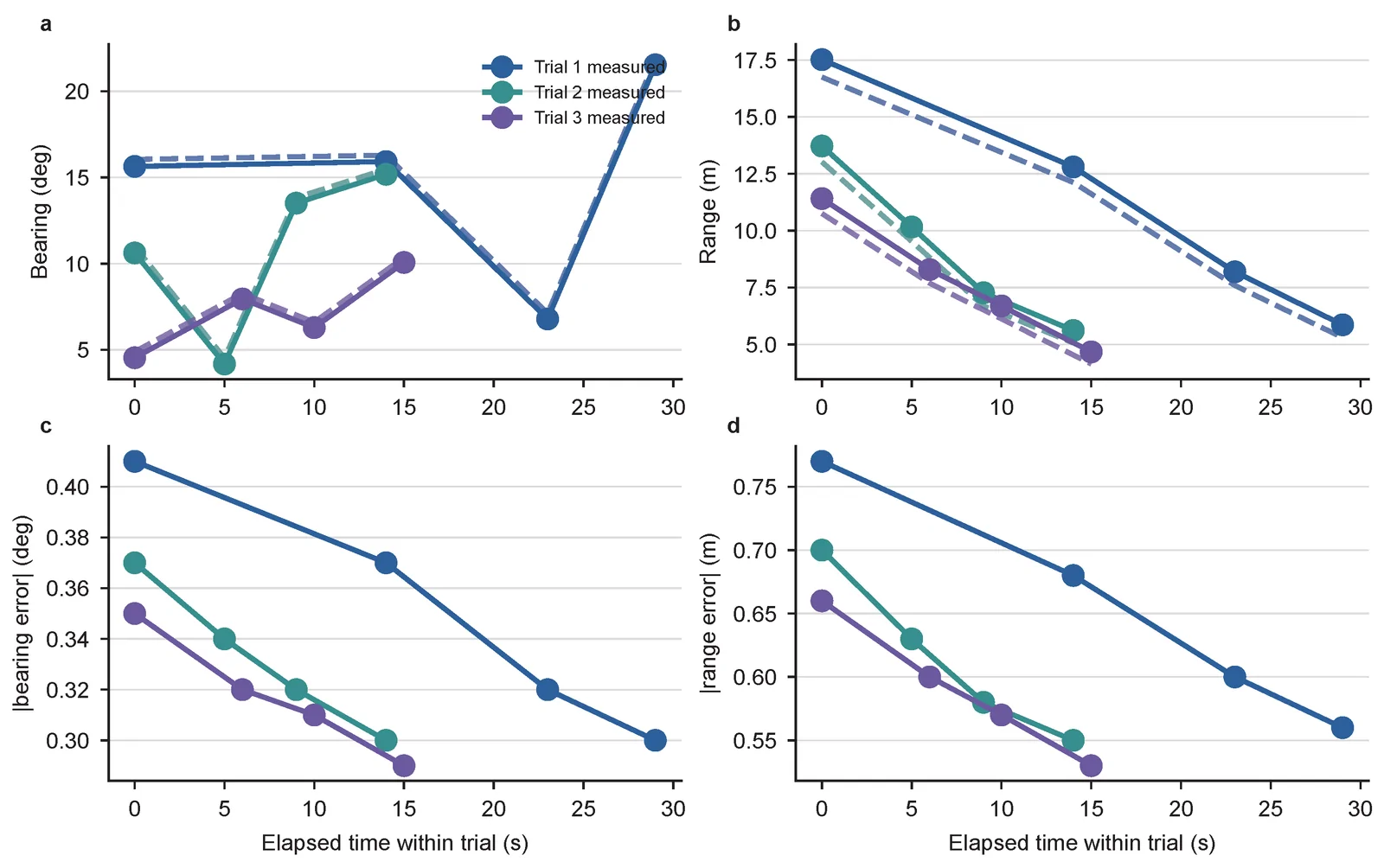
Scenario C measured overlay outputs and RTK-derived reference values computed directly from Table 11. Time is expressed relative to the first sampled frame of each lake-approach trial. (**a**) Measured and reference bearing. (**b**) Measured and reference range. In panels (**a**,**b**), solid lines with circular markers indicate the measured values, while dashed lines of the corresponding color indicate the RTK-derived reference values. Blue, teal, and purple denote Trials 1, 2, and 3, respectively. (**c**) Absolute bearing error and (**d**) absolute range error, with the three trials represented using the same blue, teal, and purple color scheme.

To analyze approach geometry under realistic wave motion, three repeated approach trials were conducted on an open lake. The USV navigated toward a floating platform equipped with an ArUco marker board. Unlike Scenarios A and B, which focused on single approach passes, Scenario C captures the interplay between the fused horizontal angle $\theta_{h}^{\mathrm{fus}}$ and the marker-normal heading $\psi_{\mathrm{yaw}}$ across multiple geometries and closing distances. The three trials represent distinct approach strategies. Trial 1 is an oblique approach with large marker-normal heading (Figure 13). Trial 2 shows moderate heading correction (Figure 14). Trial 3 is a closer-range approach in which the horizontal and vertical offsets grow again during closing (Figure 15). The sampled time-series measurements are tabulated in Table 11 and Figure 16. The table is intended as a qualitative diagnostic view; the formal docking-alignment criterion in this paper requires simultaneous convergence of $\theta_{h}^{\mathrm{fus}}$, $\psi_{\mathrm{yaw}}$, and $t_{x}^{\mathrm{fused}}$.

The three trials illustrate three distinct approach patterns, each of which VARE captures through the joint observation of $\theta_{h}^{\mathrm{fus}}$ and marker-normal heading $\psi_{\mathrm{yaw}}$:

Trial 1: Oblique approach with large bearing and heading error. Throughout the approach from 17.5 m to 5.9 m, both $\theta_{h}^{\mathrm{fus}}$ and $\psi_{\mathrm{yaw}}$ remain large ($\theta_{h}^{\mathrm{fus}}\in [6.8{}^{\circ},21.5{}^{\circ}]$, $\psi_{\mathrm{yaw}}\in [32.3{}^{\circ},45.6{}^{\circ}]$). The marker is consistently located in the right half of the image (Figure 13), and the USV is heading obliquely past the platform rather than toward it. These frames are qualitatively inconsistent with docking readiness and illustrate a clearly misdirected approach.

Trial 2: Heading correction with residual lateral offset. The $\psi_{\mathrm{yaw}}$ values are substantially smaller (7.7–13.6°) than in Trial 1, indicating that the vessel heading is closer to the marker normal. However, $\theta_{h}^{\mathrm{fus}}$ still ranges from 4.18° to 15.18°, revealing persistent lateral offset. At t=228 s, the system reaches the closest bearing-centered state ($\theta_{h}^{\mathrm{fus}}=4.18{}^{\circ}$), yet the marker-normal heading remains non-zero ($\psi_{\mathrm{yaw}}=9.74{}^{\circ}$). This trial demonstrates a heading-first correction pattern in which the vessel partially improves its pointing direction without fully removing the lateral approach bias.

Trial 3: Closing with residual heading and vertical error. In this trial, the marker-normal heading remains large and positive (20.53–27.28°) throughout the sampled frames, while the horizontal fusion angle stays in the moderate range (4.54–10.07°). As the vessel closes from $d_{xz}=11.41$ m to 4.67 m, the horizontal fusion term rises from 4.54° to 10.07°, and the overlaid vertical fusion term increases markedly from 3.90° to 15.81°. This pattern is consistent with a closing maneuver in which both lateral and vertical misalignment remain significant, again showing that proximity does not imply docking readiness.

Across all three trials (12 sampled frames), visual bearing and marker-normal heading never enter a near-zero/near-zero regime simultaneously. This is consistent with the fact that these were open-loop approach maneuvers rather than closed-loop docking runs. The closest bearing-centered condition occurs at t=228 s in Trial 2, where $\theta_{h}^{\mathrm{fus}}=4.18{}^{\circ}$ while marker-normal heading remains noticeably non-zero. A closed-loop terminal trigger should therefore require $\theta_{h}^{\mathrm{fus}}$, $\psi_{\mathrm{yaw}}$, and $t_{x}^{\mathrm{fused}}$ to satisfy their tolerance bands.

#### 4.4.5. Lateral Convergence Analysis

The docking-alignment criterion in Equation (26) requires visual angular centering, marker-normal orientation alignment, and lateral translation convergence. Since the archived sampled-field overlays do not expose frame-wise $t_{x}^{\mathrm{fused}}$, Table 10 and Table 11 report the line-of-sight proxy

(46) $$\ell_{h}=d_{xz}\sin\;\left(|\theta_{h}^{\mathrm{fus}}|\right)$$

as a conservative diagnostic for lateral convergence. The calculation converts $\theta_{h}^{\mathrm{fus}}$ to radians inside the sine. This proxy is not a replacement for $t_{x}^{\mathrm{fused}}$, and future closed-loop trials should log the actual fused lateral translation directly. It is nevertheless useful for interpreting whether a small bearing angle is operationally sufficient at the current range. In Scenario A, $\ell_{h}$ decreases from approximately 14.0 m at t=6 s to 4.8 m at t=11 s and 0.93 m at t=13 s. Even when $\theta_{h}^{\mathrm{fus}}$ is inside the 3° angular tolerance band, the implied lateral displacement remains larger than the representative $\delta_{\mathrm{tol}}=0.3$ m threshold. In Scenario C, the same proxy remains non-negligible at close range: Trial 2 gives $\ell_{h}\approx 0.74$ m at t=228 s, while Trial 3 gives $\ell_{h}\approx 0.82$ m at t=328 s. These values support the use of simultaneous bearing, heading, and translational convergence rather than a distance-only or bearing-only docking trigger.

#### 4.4.6. Static Accuracy Evaluation

Static station-keeping measurements were pooled across two physical boards at 10–40 m. Table 12 reports horizontal and vertical bearing RMSE. Marker-normal heading is excluded from the method ranking because the archived method record does not establish a method-specific heading-processing chain.

#### 4.4.7. Discussion

The field trial results yield several observations regarding the alignment criterion and the operational behavior of VARE, but they should be interpreted within the limits of the available reference information.

Static angular error. The reported independent-reference bearing reductions are 34.4–50.0% relative to the pixel-only baseline and 23.2–38.2% relative to Variant A. Marker-normal heading is not ranked across methods because the available method description supports only raw $\psi_{\mathrm{yaw}}$ extraction, not a VARE-specific heading filter that could produce method-dependent values. The raw heading values in the field overlays remain qualitative alignment diagnostics and are not used to claim method-level accuracy improvement.

Asymmetric convergence of bearing and marker-normal heading. The sampled time-series from Scenario A (Table 10) and Scenario C (Table 11) indicate that visual centering ($\theta_{h}^{\mathrm{fus}}\to 0$) and marker-normal heading correction ($\psi_{\mathrm{yaw}}\to 0$) can evolve at different rates and exhibit different failure patterns. In Scenario A, visual centering enters the retrospective bearing tolerance band ($\theta_{h}^{\mathrm{fus}}=1.09{}^{\circ}$ at t=13 s) while $\psi_{\mathrm{yaw}}$ remains at 20.12°. In Scenario C Trial 3, $\theta_{h}^{\mathrm{fus}}$ increases again at close range while $\psi_{\mathrm{yaw}}$ remains above 20°. These complementary patterns show why bearing and marker-normal heading should not be collapsed into a single indicator. In the present formulation, the formal docking-alignment criterion requires simultaneous convergence of $\theta_{h}^{\mathrm{fus}}$, $\psi_{\mathrm{yaw}}$, and $t_{x}^{\mathrm{fused}}$.

Closing distance alone is insufficient in the sampled lake trials. In Trial 1, the USV reaches $d_{xz}=5.86$ m with $\theta_{h}^{\mathrm{fus}}=21.54{}^{\circ}$ and $\psi_{\mathrm{yaw}}=42.24{}^{\circ}$; in Trial 3, it closes to $d_{xz}=4.67$ m while still exhibiting $\psi_{\mathrm{yaw}}=20.53{}^{\circ}$. These examples are inconsistent with a well-aligned final approach despite the short range. They therefore support the design choice that Equation (26) should depend on fused bearing, marker-normal heading, and lateral offset rather than on distance alone. Because the sampled tables report $\ell_{h}$ rather than direct frame-wise $t_{x}^{\mathrm{fused}}$, this evidence should be read as diagnostic rather than as a closed-loop docking validation.

Dual-path fusion behavior under varying range. Comparing the diagnostic overlays in Figure 7, Figure 8 and Figure 9 shows that the fused output remains numerically consistent with its two source angles across changing river-approach geometries. At $d_{xz}=70.64$ m (Figure 7), the image-based and geometry-based horizontal angles are $\alpha_{h}=1.68{}^{\circ}$ and $\beta_{h}=0.72{}^{\circ}$, and the fused output is $\theta_{h}^{\mathrm{fus}}=0.82{}^{\circ}$. At $d_{xz}=44.15$ m (Figure 8), the two horizontal paths are $\alpha_{h}=7.03{}^{\circ}$ and $\beta_{h}=7.89{}^{\circ}$, with $\theta_{h}^{\mathrm{fus}}=7.78{}^{\circ}$. At $d_{xz}=40.11$ m (Figure 9), the horizontal paths remain close ($\alpha_{h}=5.03{}^{\circ}$, $\beta_{h}=5.38{}^{\circ}$), yielding $\theta_{h}^{\mathrm{fus}}=5.35{}^{\circ}$, while the vertical angles increase to $\alpha_{v}=4.82{}^{\circ}$, $\beta_{v}=4.58{}^{\circ}$, and $\theta_{v}^{\mathrm{fus}}=4.61{}^{\circ}$. These isolated overlays show plausible bounded fusion behavior, but they cannot reconstruct instantaneous confidence weights or signed-error correlation.

Evidence of operation across reported environments. The three scenarios span open-river haze (Scenario A), industrially cluttered harbor imagery with metallic reflections (Scenario B), and open-lake wave motion with sun glare (Scenario C). In the reported figures, detections are visible at large off-axis views ($\theta_{h}^{\mathrm{fus}}=21.12{}^{\circ}$ in Scenario B) and in 12 sampled lake frames spanning 4.67–17.52 m. These selected observations do not provide a session-level dropout statistic or survival curve and must not be extrapolated to untested severe backlighting, heavy rain, spray, saturated reflection, or rapidly changing high sea states.

#### 4.4.8. Data Accounting, Environmental Coverage, and Failure Boundaries

Table 13 and Table 14 distinguishes the preserved evaluation unit from quantities that cannot be reconstructed from the available archive. This accounting prevents nominal acquisition duration and selected field frames from being misread as exact detection statistics.

The principal expected failure modes follow directly from the processing chain. (i) A small marker footprint, blur, occlusion, or severe glare can prevent corner detection, in which case neither bearing path nor a new PnP metric anchor is available. (ii) During prolonged marker loss, $\kappa_{t}$ becomes stale and the MiDaS cue loses metric validity. (iii) Reflection or illumination change can jointly perturb the shared corners and the relative-depth ROI, so confidence weighting cannot guarantee rejection of a common-mode error. (iv) A very large but valid rapid bearing change is deliberately attenuated by Equation (39), producing lag rather than binary suppression. (v) Reuse of an asynchronous MiDaS result can create temporal mismatch when data age is large. These are mechanism-based operating boundaries; the archive does not support occurrence rates, longest-loss statistics, or a claim that a confidence rule was independently validated under each severe condition. The oblique lake approaches in Figure 13, Figure 14 and Figure 15 illustrate unsafe approach geometry, not estimator-failure events.

### 4.5. Ablation Studies

To quantify the individual contribution of each proposed component, we conducted systematic ablation experiments using the pool-based dataset (Section 4.3), where the controlled environment permits repeated, comparable trials.

#### 4.5.1. Conventional Baseline, Simple-Smoother Controls, and Component-Level Ablation

To address the concern that the reported gains might be attributable to temporal smoothing rather than to MiDaS-anchored depth per se, the ablation in Table 15 includes three additional simple-smoother references on the *same* PnP depth stream: a first-order exponential moving average (EMA), a first-order low-pass filter, and a One-Euro filter [37]. These references isolate the information contribution of MiDaS above and beyond a lightweight temporal smoother applied directly to $t_{z}$. The optional IMU path of Section 3.9 is not exercised by the main VARE configuration and is therefore excluded from the quantitative ranking in Table 15.

Dual-path bearing fusion. Removing the dual-path module changes the reported bearing RMSE from 0.46° to 0.69°. Using the ablated configuration as the denominator, the full configuration reduces bearing RMSE by 33.3%. It is also 35.2% lower than Variant A and 47.7% lower than the pixel-only baseline under the same convention. These comparisons associate the dual-path module with lower error on the pool data. Because the number of approach trials is limited, the result is interpreted as a trial-level experimental comparison rather than as a broad distribution-level claim across vessel types, sea states, and docking geometries.

MiDaS relative to simple smoothers. Removing MiDaS increases the reported range MAE from 0.82 to 1.05 m and range RMSE from 0.90 to 1.16 m. The One-Euro control reaches 0.87 m and 0.96 m on the same aggregate table. Relative to that strongest smoother-only control, VARE reduces range MAE by 5.7% and range RMSE by 6.3%. Relative to the IPPE-square + One-Euro geometry baseline, the corresponding reductions are 4.7% and 5.3%. The incremental benefit is therefore smaller than the comparison with unsmoothed PnP. The table should be interpreted as evidence for a modest RTK-referenced range-error reduction. Because metric scale is still anchored by PnP, the claim is range stabilization under the tested maritime perception pipeline rather than independent monocular metric-depth estimation.

Adaptive filtering. Removing innovation-adaptive covariance changes bearing RMSE from 0.46° to 0.48°, so the full configuration is 4.2% lower than this ablation. The state update in Equations (33)–(38) is angular and contains no binary range gate. Range values formerly shown in this ablation row have therefore been removed rather than attributed to the bearing filter.

Optional IMU extension. Quaternion SLERP fusion is retained only as an implementation option for future rough-water tests. Because the present archive does not include a complete IMU sensor specification, camera–IMU extrinsic calibration, synchronization record, or IMU trial protocol, IMU-assisted numbers are deliberately excluded from the main ablation table and from all quantitative claims.

Incremental contribution and component interaction. Table 16 uses one percentage convention throughout: reduction by VARE relative to the compared row. The one-at-a-time comparisons show the largest bearing increment for dual-path fusion, a substantial range increment relative to removing MiDaS, and a smaller bearing increment for innovation-adaptive covariance. The gains over the two strongest smoother/geometry controls are more modest. These results demonstrate measurable incremental contributions, not a factorial interaction or super-additive synergy. The documented equations compute $\beta_{h}$ and $\beta_{v}$ from the raw center-origin PnP vector, so the MiDaS branch does not rotate the geometry-domain bearing; it can affect the final fused bearing only through $q_{z}$ and $z_{\mathrm{conf}}$ in Equations (28)–(30).

#### 4.5.2. Sensitivity to Depth Fusion Weight $\alpha_{z}$

**Figure 17:**
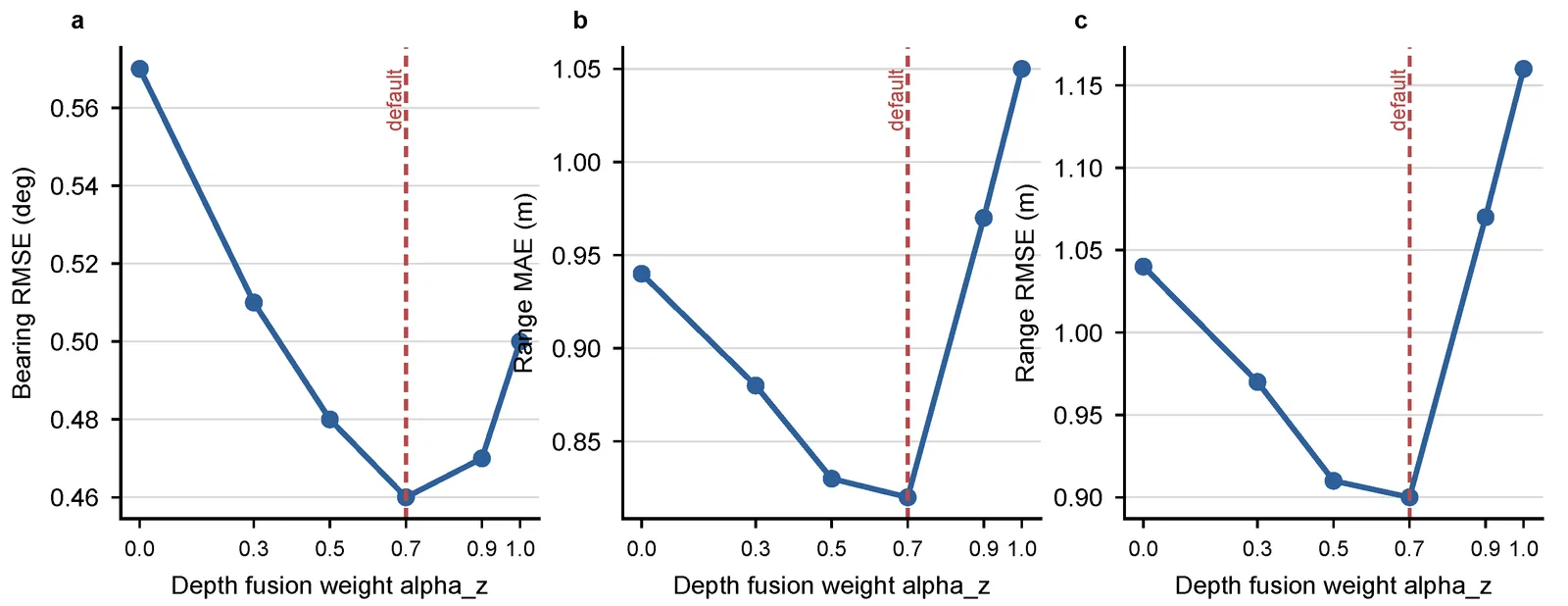
Sensitivity of VARE to the depth fusion weight $\alpha_{z}$, computed directly from Table 17. The default $\alpha_{z}=0.7$ is indicated by the vertical dashed line. Bearing and range-error metrics are separated into individual panels.

The depth fusion weight $\alpha_{z}$ (Equation (22)) is the most critical continuous hyperparameter, controlling the balance between PnP geometric depth and MiDaS learning-based depth. To assess sensitivity, we swept $\alpha_{z}$ from 0.0 (pure MiDaS) to 1.0 (pure PnP) on the pool dataset and report representative values in Table 17 and Figure 17.

The reported range metrics are lowest at $\alpha_{z}=0.5$–0.7 and degrade as the fusion approaches the PnP-only endpoint. At $\alpha_{z}=0$, the output still depends on PnP through the alignment factor $\kappa_{t}$; it is therefore not a metric-depth estimate independent of geometry. The bearing-RMSE changes across this sweep are consistent with the use of $t_{z}^{\mathrm{fused}}$ as $z_{\mathrm{conf}}$ and the continuous depth-consistency factor $q_{z}$. The sensitivity study supports the default mixed PnP–MiDaS operating region, but it does not substitute for the unavailable factorial performance sweep of the remaining confidence-shaping parameters.

## 5. Discussion and Limitations

The experiments support the separation of bearing and range channels. Land and static-water tables consistently associate VARE with lower bearing RMSE than the reported internal baselines. Pool data show lower RTK-referenced range error; however, the strongest simple smoother and the IPPE-square + One-Euro baseline approach the VARE range-error value. The field overlays add a different insight: image centering, marker-normal heading, and lateral translation are not interchangeable, and short range alone does not indicate docking readiness. This insight is operationally useful, but the present manuscript stops short of showing that it reduces recovery failure in closed-loop operation.

Validation scope. PnP returns a 6-DoF pose, but the study quantitatively validates bearing and RTK-referenced horizontal approach range. Marker-normal heading is a raw qualitative diagnostic rather than a ranked method output. The study does not report synchronized errors for $t_{x}$, $t_{y}$, roll, pitch, or full trajectories. The sampled field tables use the proxy $\ell_{h}$ because frame-wise $t_{x}^{\mathrm{fused}}$ was not available in the archived overlays. The range metrics are summarized at aggregate and trial levels, but they do not form a public frame-level benchmark.

No closed-loop docking validation. Equation (26) defines a candidate controller input condition. The trials do not report autonomous trigger success, terminal error, capture success, or failure rates. VARE is therefore evaluated as a perception module, not an end-to-end recovery system. Demonstrating task-level significance will require controller-in-the-loop or closed-loop trials that log terminal error, capture outcome, aborts, and recovery from marker loss.

Heuristic confidence weights. The confidence weights in Section 3.7 rely on marker eccentricity, PnP–MiDaS depth consistency, and nominal depth proximity. Their decay-scale and floor effects are now stated analytically, and $\alpha_{z}$ is evaluated empirically; however, the study lacks a factorial trial-level performance sweep of $z_{\mathrm{ref}}$, $\tau_{z}$, $\tau_{r}$, and the image-weight floor. The rule also omits reprojection error, marker image area, blur, per-corner re-detection success, and illumination cues. A calibrated probabilistic confidence model and a preregistered parameter sweep are natural next steps.

MiDaS limits. The relative-depth branch depends on the PnP geometry anchor and has environmental limits. Simple smoothers reproduce part of the range-error reduction, and the improvement relative to the strongest matched smoother is modest. The scale factor $\kappa_{t}$ becomes stale during prolonged PnP loss. Moreover, severe backlighting, rain or spray, saturated reflection, and rapidly changing high sea states were not tested in controlled strata. MiDaS is therefore interpreted as an auxiliary stabilizer under the reported mild-to-moderate conditions, not as an independently robust metric sensor.

Baselines are matched but not exhaustive. The comparisons include a pixel-only reference, three simple-smoother controls, internal ablations, and a matched IPPE-square + One-Euro geometry baseline. They do not include VIO-based pipelines, AprilTag-board pose stacks, learning-assisted pose refinement, contemporary cooperative-marker docking stacks, or more recent metric-depth alternatives. Those systems require sensors, target imagery, training choices, or outputs not preserved in the present archive. The reported comparison therefore supports a bounded within-dataset systems claim, not state-of-the-art dominance.

Component coupling and implementation transparency. The final bearing stream contains raw center-origin PnP bearing, PnP–MiDaS depth-consistency weighting through $q_{z}$, depth-dependent confidence weighting through $z_{\mathrm{conf}}$, and continuous innovation-adaptive covariance. The first two estimators share detected corners and are statistically dependent. No binary innovation gate or range hold is part of the evaluated formulation. Frame-level logs and evaluation scripts remain necessary to audit signed-error correlation, data age, and component interactions independently.

Statistical and failure reporting. Trial-level summaries and confidence intervals for each VARE mean are reported for the pool experiment, but paired-difference confidence intervals and independently verifiable hypothesis-test records are unavailable. The manuscript also lacks exact frame denominators, distance-binned detection rates, longest-loss runs, and empirical failure frequencies. Table 13 states these boundaries explicitly; future release of frame-wise RTK, detection, and timestamp logs is required for a reusable benchmark.

## 6. Conclusions

VARE separates image-domain bearing, PnP geometry, relative-depth stabilization, and innovation-adaptive temporal smoothing within one USV recovery-perception pipeline. The reported tests associate the full configuration with lower bearing RMSE than the pixel-only, static-PnP, and matched IPPE-square references. Pool results also show lower range MAE and RMSE, although simple temporal smoothers recover much of this benefit and the remaining improvement over the strongest matched baseline is modest.

Field overlays show that bearing, marker-normal heading, lateral displacement, and closing range can evolve differently. A recovery controller should therefore avoid a distance-only or bearing-only trigger and should use direct lateral translation rather than the proxy analyzed here. The present evidence supports VARE as a candidate perception module with independently referenced bearing and range evaluation. It does not establish full 6-DoF accuracy, session-level robustness, or closed-loop docking reliability.

The next evaluation should release frame-level logs and code; quantify signed-error covariance, MiDaS data age, detection survival, and paired trial differences; sweep all confidence-shaping parameters; and compare against task-matched AprilTag-board, visual–inertial, learning-refinement, and metric-depth baselines. Closed-loop trials should then measure terminal error, marker dropout and recovery, capture success, and failure cases across controlled sea states and illumination conditions. 

## Figures and Tables

**Table 1:** Primary reported quantities and their operational meaning.

Symbol	Interpretation	Role in This Paper
$t={\left[t_{x},t_{y},t_{z}\right]}^{⊤}$	Camera-frame metric translation of the marker center estimated by PnP	Metric geometry anchor
$\alpha_{h},\alpha_{v}$	Bearing from image-plane marker offsets relative to the principal point	Image-domain angle path
$\beta_{h},\beta_{v}$	Bearing from the center-origin PnP translation vector $\left(t_{x},t_{y},t_{z}\right)$	Geometry-domain angle path
$\theta_{h}^{\mathrm{fus}},\theta_{v}^{\mathrm{fus}}$	Confidence-weighted fusion of the two bearing paths	Main reported bearing outputs
$\psi_{\mathrm{yaw}}$	Raw marker-normal heading from the projected marker normal in the camera frame	Qualitative orientation-alignment diagnostic; not included in method ranking
$t_{z}$	PnP optical-axis metric depth	Metric anchor for MiDaS alignment
${\tilde{d}}_{\mathrm{rel}}$	Scale-invariant MiDaS ROI statistic	Relative-depth cue before metric alignment
$d_{\mathrm{midas}}$	PnP-aligned MiDaS relative-depth cue	Auxiliary range-stabilization cue
$t^{\mathrm{fused}}$	Stabilized camera-frame metric translation vector	Source vector for reported lateral translation and distances
$t_{x}^{\mathrm{fused}}$	Lateral component of the fused translation vector	Lateral-position alignment variable
$t_{z}^{\mathrm{fused}}$	Fused optical-axis metric depth	Depth channel
$d_{xyz}$	Three-dimensional slant distance derived from $t^{\mathrm{fused}}$	Defined for completeness; not the primary metric
$d_{xz}$	Horizontal-plane approach distance derived from the fused translation	Reported range metric

**Table 2:** Deterministic equation-level sensitivity of the confidence-shaping parameters. These values are consequences of Equations (28)–(30), not additional performance experiments.

Analytical Change	Deterministic Response	Interpretation
$\tau_{z}$ or $\tau_{r}=10,15,20$ m at a 15 m discrepancy	Exponential multiplier =0.223,0.368,0.472	Larger decay scales retain geometry weight more gradually.
Image-weight floor =0.2,0.3,0.4 with $w_{\mathrm{tvec}}=1$	Minimum normalized image shares =16.7%,23.1%,28.6%	The floor prevents either bearing path from being numerically eliminated.
$z_{\mathrm{ref}}=20$ m with 5, 15, and 30 m departures at $\tau_{r}=15$ m	Range-proximity multipliers =0.717,0.368,0.135	$z_{\mathrm{ref}}$ is the center of a heuristic operating-range prior, not a measured optimum.

**Table 3:** Per-module runtime on NVIDIA Jetson AGX Xavier (30 W mode, 1920×1080 input, 30 fps). Median timings over 2000 frames; 95th-percentile in parentheses. MiDaS runs asynchronously on the GPU; all other modules run on the CPU synchronously with the camera stream.

Module	Device	Time (ms)	Rate (Hz)
ArUco detection	CPU	8.2 (10.1)	∼30
solvePnP (iterative)	CPU	1.4 (1.9)	∼30
Dual-path bearing fusion (Equations (31) and (32))	CPU	0.2 (0.3)	∼30
MiDaS DPT_Large (TensorRT FP16)	GPU (async)	93 (108)	∼10
Scale alignment + EMA (Equation (20))	CPU	0.3 (0.4)	∼30
Adaptive Kalman filter update	CPU	0.5 (0.7)	∼30
End-to-end synchronous stage (CPU only)	CPU	10.6 (12.8)	∼30
Synchronous fusion stage including non-blocking read of latest completed MiDaS cue	CPU + GPU	10.9 (13.1)	30
Sustained fused output rate	—	—	28–30

**Table 4:** Camera–marker configuration used to contextualize the field detections. Pixel footprints are pinhole-model estimates from the processed-stream field of view and marker size, and are used only as detectability support.

Item	Value	Purpose
Processed stream	1920×1080, 30 fps	Common input for detection, pose recovery, and overlay logging
Camera geometry	HFOV 58–60°; $f_{x}\approx \left(1.66–1.73\right)\times 10^{3}$ px	Supports image-angle conversion and footprint estimation
Marker board	500×500 mm; ID 0	Fiducial target used in archived field examples
Detector	OpenCV ArUco with corner refinement	Improves corner localization under small marker footprints
Estimated footprint	∼12 ×12 px at 70.64 m; ∼19–22 px at 40–44 m	Confirms physical plausibility of the reported long-range detections

**Table 5:** Hyperparameter settings for the VARE pipeline. All values were fixed across experiments.

Module	Parameter	Value	Description
MiDaS fusion	$\alpha_{z}$	0.7	PnP–MiDaS depth weight
	η	0.3	Scale factor EMA coefficient
	ϵ	$10^{-6}$	Division-by-zero guard
Angle fusion	$z_{\mathrm{ref}}$	20 m	Nominal reference depth
	$\tau_{z}$	15 m	PnP–MiDaS depth-consistency decay constant
	$\tau_{r}$	15 m	Nominal-range proximity decay constant
Kalman filter	*Q*	0.01 deg^2^	Process noise variance at the 30 fps output-smoothing stage
	$R_{\min}$	0.1 deg^2^	Minimum measurement noise
	$P_{0}$	1.0 deg^2^	Initial state covariance at the first valid detection
	γ	2.0	Innovation scaling coefficient

**Table 6:** Input-parity boundary for the comparison set. Quantitative values are reported only where observations, calibration, references, and output definitions are matched.

Comparison Family	Parity Requirement	Status in This Archive
Pixel/PnP smoothers and internal ablations	Same ArUco corners, camera model, frame set, reference construction, and metric definitions	Quantitatively reported
IPPE-square + One-Euro	Same square-marker corners, intrinsics, distortion, frames, external references, and output metrics	Quantitatively reported
AprilTag 3 or AprilTag-board stacks	Corresponding tag imagery, detector/layout definition, and task-matched target-relative outputs	Not present; no numerical claim
VINS-Mono, MSCKF, or ORB-SLAM3	Synchronized inertial data, camera–IMU extrinsics, and a mapping from ego-motion to the cooperative target-relative task	Not present; no numerical claim
Learning-assisted pose refinement	Specified model, training domain, weights, and matched maritime evaluation protocol	Not present; no numerical claim

**Table 7:** Independent-reference land-test angular RMSE (deg). Measurement values were computed from the image/PnP pipeline and compared with externally measured reference geometry. H/V denotes horizontal/vertical RMSE; a single entry indicates that only the horizontal value was archived for that row.

Marker	Dist. (m)	Sweeps	Pixel (H/V)	Variant A (H/V)	VARE (H/V)
Marker 1	10	10	0.72/0.74	0.58/0.61	**0.38/0.40**
	20	11	0.80	0.66	**0.46**
	30	11	0.89	0.74	**0.55**
	40	15	0.97/0.95	0.82/0.80	**0.64/0.62**
Marker 2	10	10	0.70/0.69	0.56/0.55	**0.36/0.35**
	20	11	0.78	0.64	**0.44**
	30	11	0.87	0.72	**0.53**
	40	15	0.95/0.93	0.80/0.78	**0.62/0.60**

**Table 8:** Pool-based model experiment: independent-reference bearing and range errors for the pixel-only baseline, two internal variants, the IPPE-square geometry baseline, and VARE. Measurements were compared with synchronized external reference values. Metrics are averaged over N=15 approach trials. Best values are shown in **bold**.

Method	Bearing RMSE (deg)	Range MAE (m)	Range RMSE (m)
Pixel-only baseline (monocular)	0.88	1.34	1.47
Variant A (PnP + static KF)	0.71	1.12	1.23
Variant B (PnP + direct MiDaS substitution)	0.66	0.98	1.08
IPPE-square + One-Euro baseline	0.54	0.86	0.95
**VARE (proposed)**	**0.46**	**0.82**	**0.90**

**Table 9:** Trial-level pool statistics over N=15 approach trials. Values are first summarized at the approach-trial level and then reported as mean ± standard deviation.

Method	θ RMSE (deg)	Range MAE (m)	Range RMSE (m)
Pixel-only	0.88±0.12	1.34±0.21	1.47±0.24
Variant A	0.71±0.10	1.12±0.18	1.23±0.20
Variant B	0.66±0.09	0.98±0.15	1.08±0.17
IPPE + One-Euro	0.54±0.08	0.86±0.13	0.95±0.15
**VARE**	0.46±0.07	0.82±0.12	0.90±0.14

*Note:* Variant A denotes PnP with a static Kalman filter; Variant B denotes PnP with direct MiDaS depth stabilization. IPPE + One-Euro denotes the square-marker IPPE pose baseline followed by One-Euro temporal smoothing. Hypothesis-test *p* values are not reported because the archived materials do not preserve the named test, paired trial differences, or analysis record needed for independent verification.

**Table 10:** Scenario A measured overlay outputs and RTK-derived reference values for the representative open-loop observation sequence. The experimental values are the image-overlay readouts. Reference values were computed from synchronized external geometry; $e_{\theta }$ and $e_{d}$ are absolute errors.

*t* (s)	$\theta_{h}^{\mathrm{fus}}$ Meas.	$\theta_{h}$ Ref.	$e_{\theta }$	$d_{\mathrm{xz}}$ Meas.	$d_{\mathrm{xz}}$ Ref.	$e_{d}$	$\psi_{\mathrm{yaw}}$
	(deg)	(deg)	(deg)	(m)	(m)	(m)	(deg)
6	17.58	18.25	0.67	46.49	45.20	1.29	29.38
11	5.86	6.53	0.67	47.10	45.80	1.30	27.46
13	1.09	1.78	0.69	48.70	47.37	1.33	20.12
87	2.40	3.08	0.68	47.63	46.32	1.31	17.04

**Table 11:** Scenario C measured overlay outputs and RTK-derived reference values from three lake platform approach trials. Experimental values are retained from the image overlays. Reference values were computed from synchronized external geometry after lever-arm correction.

Trial	*t* (s)	$\theta_{h}^{\mathrm{fus}}$ Meas.	$\theta_{h}$ Ref.	$e_{\theta }$	$d_{\mathrm{xz}}$ Meas.	$d_{\mathrm{xz}}$ Ref.	$e_{d}$	$\psi_{\mathrm{yaw}}$
		(deg)	(deg)	(deg)	(m)	(m)	(m)	(deg)
1	132	15.64	16.05	0.41	17.52	16.75	0.77	41.77
1	146	15.92	16.29	0.37	12.80	12.12	0.68	45.63
1	155	6.79	7.11	0.32	8.19	7.59	0.60	32.34
1	161	21.54	21.84	0.30	5.86	5.30	0.56	42.24
2	223	10.62	10.99	0.37	13.72	13.02	0.70	7.74
2	228	4.18	4.52	0.34	10.16	9.53	0.63	9.74
2	232	13.51	13.83	0.32	7.27	6.69	0.58	13.55
2	237	15.18	15.48	0.30	5.61	5.06	0.55	11.97
3	313	4.54	4.89	0.35	11.41	10.75	0.66	27.28
3	319	7.95	8.27	0.32	8.29	7.69	0.60	24.96
3	323	6.29	6.60	0.31	6.69	6.12	0.57	21.58
3	328	10.07	10.36	0.29	4.67	4.14	0.53	20.53

**Table 12:** Near-shore static station-keeping bearing RMSE (deg), pooled over two boards and four distances (N=8 board–distance groups). Errors are computed against independent external reference geometry. H: horizontal bearing; V: vertical bearing.

Marker Set	Dist. (m)	Pixel Baseline		Variant A		VARE	
		H	V	H	V	H	V
Pooled over two boards	10	0.72	0.68	0.58	0.55	**0.36**	**0.34**
	20	0.80	0.76	0.66	0.62	**0.45**	**0.42**
	30	0.88	0.84	0.74	0.70	**0.54**	**0.50**
	40	0.96	0.92	0.82	0.78	**0.63**	**0.58**

**Table 13:** Preserved evaluation units and reporting boundaries. “Nominal” denotes an acquisition-scale estimate, not an exact detected-frame count.

Campaign	Preserved Evaluation Unit	Unavailable Frame-Wise Quantity
Land	Two boards, four distances, 94 measurement configurations in Table 7	Signed paired-error logs for frame-level path correlation
Pool	15 approaches, each about 30 s at 30 fps (nominally approximately 13,500 acquired frames)	Exact recorded/evaluable/detected frames, distance-binned detection rate, and consecutive-loss runs
River	Selected overlay frames, including one four-frame approach sequence	Session denominator, detection survival, and recovery time
Harbor	Two selected overlay frames	Session denominator and dropout probability
Lake	Three trials and 12 sampled time points in Table 11	Full-session detection and failure statistics

**Table 14:** Mechanism-based failure boundaries and the evidence available for each condition.

Condition	Expected Pipeline Consequence	Evidence Boundary
Small footprint, blur, occlusion, or severe glare	Corner detection can fail, removing both bearing paths and the new PnP metric anchor.	Representative images only; no distance-binned detection rate or longest-loss statistic.
Prolonged marker loss	The retained scale factor $\kappa_{t}$ becomes stale, so MiDaS no longer provides a defensible metric range cue.	Not quantified; no claim of indefinite metric tracking after loss.
Reflection or relative-depth ROI corruption	Shared corner error or erroneous $q_{z}$ can affect both paths or suppress a valid geometry contribution.	Mechanism identified, but event frequency and rejection correctness are unavailable.
Large valid rapid turn	Innovation-adaptive covariance attenuates the residual and can introduce lag.	Analytical behavior is documented; no event-level false-rejection rate.
Asynchronous depth under rapid motion	Reusing the latest MiDaS cue can create non-zero data age and temporal mismatch.	Aggregate runtime is available; frame-wise data-age distribution is not.

**Table 15:** Independent-reference ablation results on the pool-based dataset (N=15 approach trials). Best vision-only values are shown in bold. Range metrics are meter-scale errors relative to synchronized external reference values rather than frame-to-frame variation; the IPPE-square row is included as a strong planar-marker geometry baseline. The optional IMU path is excluded from this ranking. Dashes in the adaptive-filter row indicate that range-side values are not attributable to the scalar bearing filter and are therefore not ranked.

Configuration	Bearing RMSE (deg)	Range MAE (m)	Range RMSE (m)
Conventional reference, strong geometry baseline, and internal variants			
Pixel-only monocular baseline	0.88	1.34	1.47
Variant A (PnP + static KF)	0.71	1.12	1.23
Variant B (PnP + direct MiDaS)	0.66	0.98	1.08
IPPE-square + One-Euro baseline	0.54	0.86	0.95
Simple-smoother controls on PnP $t_{z}$ (no MiDaS)			
PnP + EMA	0.61	0.93	1.02
PnP + low-pass	0.59	0.91	1.00
PnP + One-Euro filter	0.56	0.87	0.96
Component-level ablation			
w/o dual-path angle fusion	0.69	0.82	0.91
w/o MiDaS depth stabilization	0.50	1.05	1.16
w/o adaptive KF	0.48	–	–
**VARE (full, vision only)**	**0.46**	**0.82**	**0.90**

**Table 16:** Descriptive error reductions of the full VARE configuration relative to each comparison row in Table 15. Each percentage uses the compared configuration as the denominator; dashes denote a metric not attributed to that component.

Compared Configuration	Bearing RMSE Reduction	Range MAE Reduction	Range RMSE Reduction
w/o dual-path angle fusion	33.3%	–	–
w/o MiDaS depth stabilization	8.0%	21.9%	22.4%
w/o adaptive KF	4.2%	–	–
PnP + One-Euro filter	17.9%	5.7%	6.3%
IPPE-square + One-Euro baseline	14.8%	4.7%	5.3%

**Table 17:** Sensitivity of VARE to the depth fusion weight $\alpha_{z}$. Metrics are independent-reference bearing and range errors averaged over N=15 pool-based approach trials. Bearing is included because $\alpha_{z}$ enters the depth-consistency and depth-dependent confidence paths through $q_{z}$ and $z_{\mathrm{conf}}$.

$\alpha_{z}$	Bearing RMSE (deg)	Range MAE (m)	Range RMSE (m)
0.0 (aligned MiDaS branch)	0.57	0.94	1.04
0.3	0.51	0.88	0.97
0.5	0.48	0.83	0.91
**0.7 (default)**	**0.46**	**0.82**	**0.90**
0.9	0.47	0.97	1.07
1.0 (pure PnP)	0.50	1.05	1.16

## Data Availability

The camera intrinsic matrix, distortion coefficients, ArUco dictionary and detector settings, acquisition settings, marker specification, synchronized RTK/reference summaries, camera/RTK/marker calibration and lever-arm records, timestamp-matching records, aggregate and trial-level metric summaries, table-level source data, aggregate runtime summaries, and representative images used in this study are available from the corresponding author at chenchen_123@stu.xidian.edu.cn upon reasonable request. A public frame-wise detection-log and evaluation-code archive is not presently available; the reporting limits created by that absence are stated in Section 4.4.8.
